# Antiparasitic Activity of Fluorophenyl-Substituted Pyrimido[1,2-*a*]benzimidazoles

**DOI:** 10.3390/biomedicines11010219

**Published:** 2023-01-14

**Authors:** Ibrahim S. Al Nasr, Waleed S. Koko, Tariq A. Khan, Rainer Schobert, Bernhard Biersack

**Affiliations:** 1 Department of Biology, College of Science and Arts, Qassim University, Unaizah 51911, Saudi Arabia; 2 Department of Science Laboratories, College of Science and Arts, Qassim University, Ar Rass 51921, Saudi Arabia; 3 Department of Clinical Nutrition, College of Applied Health Sciences, Qassim University, Ar Rass 51921, Saudi Arabia; 4 Organic Chemistry Laboratory, University Bayreuth, Universitätsstrasse 30, 95440 Bayreuth, Germany

**Keywords:** heterocycle, benzimidazole, fluorine, antiparasitic drugs, neglected tropical diseases

## Abstract

A series of fourteen pyrimido[1,2-*a*]benzimidazole compounds was prepared by straightforward heterocyclic chemistry and oxidation methods. The new pyrimidobenzimidazole derivative **2a** with a 3-fluorophenyl substituent was identified as a new antiparasitic compound showing excellent activities against *Leishmania major* parasites. **2a** was highly active against *L. major* promastigotes and amastigotes with EC_50_ values in the nanomolar concentration range. Compound **3b** was less active than **2a** against *L. major*, but more active against *Toxoplasma gondii* with considerable selectivity. Hence, two promising and selective antiparasitic drug candidates **2a** and **3b** for the treatment of two parasitic diseases were identified, which can be prepared by green chemistry methods using simple one-pot reactions and oxidation procedures, respectively.

## 1. Introduction

Heterocyclic organic compounds play a crucial role for the design of new drugs for the treatment of various human diseases. *N*-Heterocyclic drugs such as Vinca alkaloids, camptothecins and nucleotides (e.g., 5-fluorouracil) are currently applied for the treatment of cancer diseases [1]. Azacycles also perform well against numerous life-threatening infectious diseases. Ribavirin, zidovudine, remdesivir, and paxlovid are prominent examples of antiviral drugs [2]. In terms of protozoal parasite infections, quinoline alkaloids such as quinine, chloroquine, and mefloquine are applied for the treatment of malaria, while the nitroimidazole metronidazole is clinically used on patients suffering from amoebiasis or giardiasis [3,4]. Benznidazole (a drug against Chagas disease) and fexinidazol (for the treatment of sleeping sickness) are further antiparasitic imidazoles [5]. The related benzimidazole scaffold acts as an isoster of natural purine bases and is a crucial component of anthelmintic (albendazole, mebendazole) and antiviral drugs (maribavir) representing a prolific field of medicinal chemistry research [6]. Pyrimidobenzimidazoles are a class of *N*-heterocycles with fused benzimidazole and pyrimidine moieties. Members of this class exhibited antibacterial, antimalarial, anticancer, and antioxidant activities, and the synthesis of pyrimido[1,2-*a*]benzimidazole derivatives via atom economic multicomponent reactions appears to be especially promising for the design of new drug candidates against various human diseases [7].

Leishmaniasis is as a neglected tropical disease (NTD), and cutaneous leishmaniasis (CL) caused by various *Leishmania* species (e.g., *L. major*, *L. tropica*, *L. mexicana*, *L. amazonensis*, etc.) is the dominant leishmaniasis form with up to 1 million, mostly young, cases every year [8,9]. CL leads to painful and stigmatizing skin lesions, and most patients suffering from CL receive pentavalent antimonials, miltefosine, amphotericin, or pentamidine [9,10,11]. While some of these drugs are toxic (e.g., the antimonials), the formation of drug resistance poses another increasing problem, which requires the development of new antileishmanial agents. Toxoplasmosis is another neglected parasitic disease caused by apicomplexan *Toxoplasma gondii* parasites, which leads to severe infections among immune-compromised people and also demands the development of new drugs [12].

**Figure 1:**
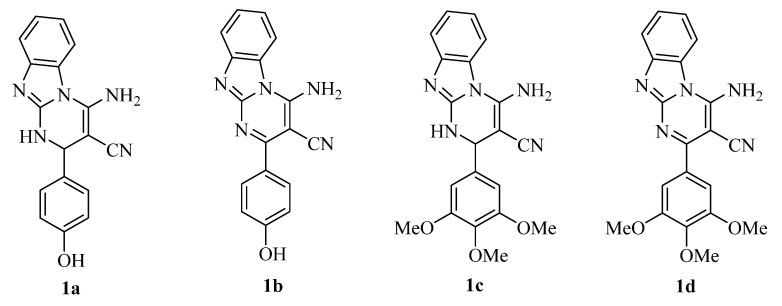
Structures of known pyrimidobenzimidazoles with documented anticancer activity.

The pyrimidobenzimidazoles **1a**–**d** were reported to possess moderate anticancer activities (Figure 1) [13]. In the present report, we disclose a series of pyrimidobenzimidazoles and their promising activities against *L. major* and *T. gondii* parasites.

## 2. Materials and Methods

### 2.1. Chemistry

The known compounds **2c**, **2d**, and **3d** were prepared following literature procedures and analyzed (see Supplementary Materials Figures S1–S33). The obtained analytical data were in line with published data of these compounds [13,14]. Synthetic procedures and analytical data of the new derivatives are given below. All starting compounds were purchased from the usual retailers and used without further purification. Column chromatography: silica gel 60 (230–400 mesh). Melting points (uncorrected), Electrothermal 9100; NMR spectra, Bruker Avance 300/500 spectrometer; chemical shifts are given in parts per million (*δ*) downfield from tetramethylsilane as internal standard; Mass spectra, Thermo Finnigan MAT 8500 (EI), UPLC/Orbitrap (ESI-HRMS).

#### 2.1.1. 4-Amino-2-(3-fluorophenyl)-1,2-dihydropyrimido[1,2-*a*]benzimidazole-3-carbonitrile (**2a**)

Malononitrile (33 mg, 0.5 mmol), 2-aminobenzimidazole (66 mg, 0.5 mmol), and 3-fluorobenzaldehyde (62 mg, 0.5 mmol) were suspended in H_2_O (5 mL) and the reaction mixture was stirred, at 80 °C, for 24 h. The formed precipitate was collected, washed with water, a small amount of ethanol and diethyl ether and dried in vacuum. Yield: 92 mg (0.30 mmol, 60%); off-white solid of m.p. 216–217 °C (dec.); IR *υ*_max_(ATR)/cm^−1^ 3457, 3325, 3223, 3057, 2883, 2190, 1679, 1639, 1601, 1489, 1467, 1443, 1402, 1246, 1212, 1162, 1136, 1104, 1030, 1004, 955, 941, 893, 795, 775, 754, 731, 714, 701, 658; ^1^H NMR (300 MHz, DMSO-d_6_) *δ* 5.28 (1 H, s), 6.89 (2 H, s), 6.9–7.1 (5 H, m), 7.1–7.2 (1 H, m), 7.3–7.4 (1 H, m), 7.63 (1 H, d, J = 7.7 Hz), 8.64 (1 H, s); ^13^C NMR (75.5 MHz, DMSO-d_6_) *δ* 52.6, 61.3, 112.5–112.9 (m), 114.5–114.8 (m), 116.1, 119.0, 120.0, 121.9, 123.4, 129.2, 130.8–131.0 (m), 143.5, 145.8, 149.3, 1516, 160.5–163.8 (m); *m/z* (%) 305 (59) [M^+^], 238 (26), 210 (100), 133 (99), 90 (43). HRMS for C_17_H_13_N_5_F [M^+^ + H] calcd. 306.11495, found 306.11460.

#### 2.1.2. 4-Amino-2-(3,5-difluorophenyl)-1,2-dihydropyrimido[1,2-*a*]benzimidazole-3-carbonitrile (**2b**)

Malononitrile (33 mg, 0.5 mmol), 2-aminobenzimidazole (66 mg, 0.5 mmol), and 3,5-difluorobenzaldehyde (71 mg, 0.5 mmol) were suspended in H_2_O (5 mL), and the reaction mixture was stirred, at 80 °C, for 24 h. The formed precipitate was collected, washed with water, a small amount of ethanol and diethyl ether, and dried in vacuum. Yield: 75 mg (0.23 mmol, 46%); off-white solid of m.p. 279–280 °C; IR *υ*_max_(ATR)/cm^−1^ 3459, 3321, 3222, 3058, 2881, 2193, 1679, 1640, 1623, 1599, 1465, 1444, 1404, 1336, 1310, 1258, 1244, 1210, 1180, 1164, 1117, 1105, 1032, 990, 974, 914, 891, 872, 846, 825, 749, 732, 717, 708, 680, 667, 630; ^1^H NMR (500 MHz, DMSO-d_6_) *δ* 5.34 (1 H, s), 6.9–7.1 (5 H, m), 7.1–7.2 (2 H, m), 7.25 (1 H, d, J = 7.8 Hz), 7.64 (1 H, d, J = 8.1 Hz), 8.68 (1 H, s); ^13^C NMR (125.8 MHz, DMSO-d_6_) *δ* 52.2, 60.4, 103.1–103.5 (m), 109.1–109.3 (m), 112.5, 116.2, 118.9, 120.0, 123.4, 129.1, 143.4, 147.5, 149.4, 151.3, 161.4, 163.4; *m/z* (%) 323 (57) [M^+^], 256 (8), 210 (100), 133 (85). HRMS for C_17_H_12_N_5_F_2_ [M^+^ + H] calcd. 324.10553, found 324.10488.

#### 2.1.3. 4-Amino-2-(3-bromo-4,5-dimethoxyphenyl)-1,2-dihydropyrimido[1,2-*a*]benzimidazole-3-carbonitrile (**2e**)

Malononitrile (33 mg, 0.5 mmol), 2-aminobenzimidazole (66 mg, 0.5 mmol), and 3-bromo-4,5-dimethoxybenzaldehyde (122 mg, 0.5 mmol) were suspended in H_2_O (10 mL), and the reaction mixture was stirred, at 80 °C, for 24 h. The formed precipitate was collected, washed with water, a small amount of ethanol and diethyl ether, and dried in vacuum. The resulting solid was recrystallized from DMSO/EtOH/toluene. Yield: 60 mg (0.14 mmol, 28%); off-white solid of m.p. 282–283 °C; IR *υ*_max_(ATR)/cm^−1^ 3438, 3328, 3223, 3003, 2935, 2834, 2184, 1675, 1637, 1602, 1572, 1489, 1473, 1464, 1444, 1428, 1400, 1337, 1287, 1269, 1258, 1234, 1178, 1163, 1141, 1103, 1046, 1002, 965, 915, 890, 881, 864, 804, 781, 748, 734, 713, 691, 662, 641, 617, 607; ^1^H NMR (500 MHz, DMSO-d_6_) *δ* 3.70 (3 H, s), 3.77 (3 H, s), 5.24 (1 H, s), 6.92 (2 H, s), 6.9–7.0 (2 H, m), 7.06 (1 H, s), 7.1–7.2 (1 H, m), 7.24 (1 H, d, J = 7.8 Hz), 7.64 (1 H, d, J = 8.1 Hz), 8.59 (1 H, s); ^13^C NMR (125.8 MHz, DMSO-d_6_) *δ* 52.4, 56.0, 60.1, 60.9, 110.7, 112.5, 116.1, 116.6, 119.1, 120.0, 121.4, 123.5, 129.2, 140.2, 143.5, 145.2, 149.4, 151.5, 153.5; *m/z* (%) 427 (28) [M^+^], 425 (30) [M^+^], 210 (73), 133 (100). HRMS for C_19_H_17_N_5_O_2_^79^Br [M^+^ + H] calcd. 426.05601, found 426.05517, for C_19_H_17_N_5_O_2_^81^Br [M^+^ + H] calcd. 428.05397, found 428.05306.

#### 2.1.4. 4-Amino-2-(3-pyridyl)-1,2-dihydropyrimido[1,2-*a*]benzimidazole-3-carbonitrile (**2f**)

Malononitrile (33 mg, 0.5 mmol), 2-aminobenzimidazole (66 mg, 0.5 mmol), and pyridine-3-carboxaldehyde (54 mg, 0.5 mmol) were suspended in H_2_O (5 mL), and the reaction mixture was stirred, at 80 °C, for 24 h. The formed precipitate was collected, washed with water, a small amount of ethanol and diethyl ether, and dried in vacuum. Yield: 98 mg (0.34 mmol, 68%); off-white solid of m.p. 289–290 °C; IR *υ*_max_(ATR)/cm^−1^ 3467, 3303, 3058, 3019, 2922, 2183, 1663, 1643, 1606, 1585, 1448, 1472, 1434, 1405, 1355, 1319, 1285, 1262, 1246, 1205, 1161, 1149, 1131, 1104, 1070, 1030, 968, 918, 891, 845, 814, 752, 733, 708, 666, 639, 630, 618; ^1^H NMR (500 MHz, DMSO-d_6_) *δ* 5.35 (1 H, s), 6.92 (2 H, s), 7.0–7.1 (1 H, m), 7.1–7.2 (1 H, m), 7.24 (1 H, d, J = 7.8 Hz), 7.4–7.5 (1 H, m), 7.6–7.7 (2 H, m), 8.5–8.6 (2 H, m), 8.60 (1 H, s); ^13^C NMR (125.8 MHz, DMSO-d_6_) *δ* 51.2, 61.0, 112.5, 116.2, 118.8, 120.0, 123.4, 123.8, 129.2, 133.9, 138.0, 143.5, 147.6, 149.2, 149.4, 151.6; *m/z* (%) 288 (72) [M^+^], 221 (21), 210 (100), 133 (96). HRMS for C_16_H_13_N_6_ [M^+^ + H] calcd. 289.11962, found 289.11889.

#### 2.1.5. 4-Amino-2-(3-fluorophenyl)pyrimido[1,2-*a*]benzimidazole-3-carbonitrile (**3a**)

**2a** (25 mg, 0.082 mmol) was dissolved in hot DMF (1 mL), and *p*-chloranil (21 mg, 0.085 mmol) was added. The reaction mixture was stirred, at 120 °C, for 5 min. H_2_O (0.1 mL) was added, and the reaction mixture was stirred, at 120 °C, for 3 min. After cooling to room temperature, the formed precipitate was collected and washed with ethanol. Yield: 21 mg (0.069 mmol, 84%); yellow solid of m.p. > 370 °C (dec.); IR *υ*_max_(ATR)/cm^−1^ 3452, 3393, 2561, 2208, 1664, 1635, 1615, 1574, 1552, 1497, 1489, 1474, 1450, 1385, 1313, 1271, 1263, 1230, 1184, 1166, 1149, 1133, 1099, 1063, 1004, 966, 904, 882, 850, 815, 786, 760, 740, 726, 714, 682, 666; ^1^H NMR (500 MHz, DMSO-d_6_) *δ* 7.3–7.5 (2 H, m), 7.5–7.7 (3 H, m), 7.7–7.8 (2 H, m), 8.60 (1 H, d, J = 8.3 Hz), 8.79 (2 H, br s); ^13^C NMR (125.8 MHz, DMSO-d_6_) *δ* 115.0–115.3 (m), 115.6, 116.6, 117.3–117.5 (m), 121.8, 124.9–125.0 (m), 126.3–126.4 (m), 130.5–130.6 (m), 139.4, 150.4, 154.5, 160.1–163.3 (m), 162.3; HRMS for C_17_H_11_N_5_F [M^+^ + H] calcd. 304.09930, found 304.09842.

#### 2.1.6. 4-Amino-2-(3,5-difluorophenyl)pyrimido[1,2-*a*]benzimidazole-3-carbonitrile (**3b**)

**2b** (27 mg, 0.082 mmol) was dissolved in hot DMF (1 mL), and *p*-chloranil (21 mg, 0.085 mmol) was added. The reaction mixture was stirred, at 120 °C, for 5 min. H_2_O (0.1 mL) was added, and the reaction mixture was stirred, at 120 °C, for 3 min. After cooling to room temperature, the formed precipitate was collected and washed with ethanol. Yield: 25 mg (0.078 mmol, 95%); yellow solid of m.p. > 370 °C (dec.); IR *υ*_max_(ATR)/cm^−1^ 3295, 3223, 3072, 2213, 1638, 1601, 1574, 1481, 1446, 1413, 1387, 1343, 1308, 1289, 1266, 1179, 1143, 1128, 1014, 993, 901, 885, 866, 815, 791, 766, 757, 746, 723, 684, 663; ^1^H NMR (500 MHz, DMSO-d_6_) *δ* 7.3–7.5 (1 H, m), 7.5–7.7 (4 H, m), 7.78 (1 H, d, J = 8.0 Hz), 8.60 (1 H, d, J = 8.3 Hz), 8.83 (2 H, br s); ^13^C NMR (125.8 MHz, DMSO-d_6_) *δ* 105.6–106.3 (m), 111.9–112.3 (m), 115.1, 116.4, 121.9, 126.3, 140.5, 150.2, 154.4, 160.3, 160.5, 161.2–163.7 (m), 163.6; HRMS for C_17_H_10_N_5_F_2_ [M^+^ + H] calcd. 322.08988, found 322.08897.

#### 2.1.7. 4-Amino-2-(2-fluorophenyl)pyrimido[1,2-*a*]benzimidazole-3-carbonitrile (**3c**)

**2c** (25 mg, 0.082 mmol) was dissolved in hot DMF (1 mL), and *p*-chloranil (21 mg, 0.085 mmol) was added. The reaction mixture was stirred, at 120 °C, for 5 min. H_2_O (0.1 mL) was added and the reaction mixture was stirred, at 120 °C, for 3 min. After cooling to room temperature, the formed precipitate was collected and washed with ethanol. Yield: 15 mg (0.05 mmol, 61%); off-white solid of m.p. > 350 °C (dec.); IR *υ*_max_(ATR)/cm^−1^ 3175, 2219, 1644, 1607, 1570, 1491, 1453, 1420, 1394, 1265, 1216, 1141, 1102, 813, 756, 736, 681, 660; ^1^H NMR (500 MHz, DMSO-d_6_) *δ* 7.3–7.5 (3 H, m), 7.5–7.7 (3 H, m), 7.79 (1 H, d, J = 8.1 Hz), 8.61 (1 H, d, J = 8.3 Hz), 8.84 (2 H, br s); ^13^C NMR (125.8 MHz, DMSO-d_6_) *δ* 115.0, 115.8–116.1 (m), 118.8, 121.8, 124.7, 125.5, 126.2–126.4 (m), 130.9, 132.3–132.4 (m), 153.8, 157.3- 160.6 (m), 160.4; HRMS for C_17_H_11_N_5_F [M^+^ + H] calcd. 304.09930, found 304.09856.

#### 2.1.8. 4-Pyrrolo-2-(3-fluorophenyl)-1,2-dihydropyrimido[1,2-*a*]benzimidazole-3-carbonitrile (**4a**)

**2a** (85 mg, 0.28 mmol) and 2,5-dimethoxytetrahydrofuran (58 µL, 0.46 mmol) were suspended in acetic acid (5 mL). The reaction mixture was stirred, at 90 °C, for 3 h. After cooling down, water was added, and the formed precipitate was collected, washed with water, and dried in vacuum. Yield: 38 mg (0.11 mmol, 39%); brown solid of m.p. > 260 °C (dec.); IR *υ*_max_(ATR)/cm^−1^ 3118, 2875, 2216, 1668, 1586, 1458, 1403, 1381, 1333, 1305, 1261, 1217, 1163, 1139, 1091, 1074, 1012, 977, 954, 893, 765, 755, 741, 722, 696; ^1^H NMR (500 MHz, DMSO-d_6_) *δ* 5.32 (1 H, d, J = 7.6 Hz), 5.80 (1 H, s), 6.4–6.5 (2 H, m), 6.7–6.8 (1 H, m), 7.0–7.1 (1 H, m), 7.2–7.4 (4 H, m), 7.4–7.5 (2 H, m), 7.5–7.6 (1 H, m), 8.8–9.0 (1 H, br s); ^13^C NMR (125.8 MHz, DMSO-d_6_) *δ* 54.3, 88.4, 110.1, 111.2, 113.8, 114.1, 114.8, 115.5, 115.8, 116.2, 120.4, 122.0–122.3 (m), 123.0, 123.7, 128.5, 131.1–131.2 (m), 141.6, 143.3–143.4 (m), 150.5, 160.7–164.0 (m); HRMS for C_21_H_15_N_5_F [M^+^ + H] calcd. 356.13060, found 356.12978.

#### 2.1.9. 4-Pyrrolo-2-(3,5-difluorophenyl)-1,2-dihydropyrimido[1,2-*a*]benzimidazole-3-carbonitrile (**4b**)

**2b** (65 mg, 0.20 mmol) and 2,5-dimethoxytetrahydrofuran (41 µL, 0.33 mmol) were suspended in acetic acid (5 mL). The reaction mixture was stirred, at 90 °C, for 3 h. After cooling down, water was added, and the formed precipitate was collected, washed with water, and dried in vacuum. Yield: 22 mg (0.059 mmol, 30%); brown solid of m.p. > 240 °C; IR *υ*_max_(ATR)/cm^−1^ 3093, 2873, 2215, 1669, 1623, 1599, 1459, 1405, 1339, 1216, 1123, 990, 961, 862, 757, 723; ^1^H NMR (500 MHz, DMSO-d_6_) *δ* 5.32 (1 H, d, J = 7.8 Hz), 5.85 (1 H, s), 6.4–6.5 (2 H, m), 6.7–6.8 (1 H, m), 7.0–7.1 (1 H, m), 7.1–7.4 (6 H, m), 8.8–9.0 (1 H, br s); ^13^C NMR (125.8 MHz, DMSO-d_6_) *δ* 54.1, 87.8, 104.0–104.7 (m), 110.2–111.1 (m), 114.7, 116.2, 120.5, 121.9, 122.4, 123.8, 128.6, 141.9, 143.2, 144.9–145.1 (m), 150.2, 160.8–164.3 (m); HRMS for C_21_H_14_N_5_F_2_ [M^+^ + H] calcd. 374.12118, found 374.12023.

#### 2.1.10. 4-Pyrrolo-2-(2-fluorophenyl)-1,2-dihydropyrimido[1,2-*a*]benzimidazole-3-carbonitrile (**4c**)

**2c** (100 mg, 0.33 mmol) and 2,5-dimethoxytetrahydrofuran (69 µL, 0.55 mmol) were suspended in acetic acid (5 mL). The reaction mixture was stirred, at 90 °C, for 3 h. After cooling down, water was added, and the formed precipitate was collected, washed with water, and dried in vacuum. Yield: 30 mg (0.084 mmol, 26%); brown solid of m.p. > 240 °C (dec.); IR *υ*_max_(ATR)/cm^−1^ 3132, 2872, 2217, 1675, 1584, 1461, 1461, 1425, 1379, 1268, 1248, 1227, 1204, 1152, 1095, 10734, 1014, 972, 901, 851, 829, 757, 734; ^1^H NMR (500 MHz, DMSO-d_6_) *δ* 5.31 (1 H, d, J = 7.9 Hz), 5.95 (1 H, s), 6.4–6.5 (2 H, m), 6.7–6.8 (1 H, m), 7.0–7.1 (1 H, m), 7.2–7.4 (5 H, m), 7.4–7.5 (1 H, m), 7.5–7.6 (1 H, m), 8.7–8.9 (1 H, br s); ^13^C NMR (125.8 MHz, DMSO-d_6_) *δ* 50.2, 87.6, 110.0, 111.3, 114.6, 115.9, 116.2, 120.4, 122.0, 123.7, 125.1–125.2 (m), 127.2–127.3 (m), 128.5, 129.3–129.4 (m), 131.0, 141.7, 143.3, 150.6, 158.4–161.6 (m); HRMS for C_21_H_15_N_5_F [M^+^ + H] calcd. 356.13060, found 356.12969.

#### 2.1.11. 4-Pyrrolo-2-(4-fluorophenyl)-1,2-dihydropyrimido[1,2-*a*]benzimidazole-3-carbonitrile (**4d**)

**2d** (78 mg, 0.26 mmol) and 2,5-dimethoxytetrahydrofuran (54 µL, 0.43 mmol) were suspended in acetic acid (5 mL). The reaction mixture was stirred, at 90 °C, for 3 h. After cooling down, water was added, and the formed precipitate was collected, washed with water, and dried in vacuum. Yield: 25 mg (0.07 mmol, 27%); brown solid of m.p. > 260 °C; IR *υ*_max_(ATR)/cm^−1^ 3128, 2848, 2217, 1672, 1601, 1509, 1460, 1403, 1273, 1228, 1158, 1095, 1013, 974, 900, 886, 839, 787, 753, 725, 697; ^1^H NMR (500 MHz, DMSO-d_6_) *δ* 5.31 (1 H, d, J = 8.0 Hz), 5.76 (1 H, s), 6.4–6.5 (2 H, m), 6.7–6.8 (1 H, m), 7.0–7.1 (1 H, m), 7.2–7.4 (5 H, m), 7.5–7.6 (2 H, m), 8.7–8.9 (1 H, br s); ^13^C NMR (125.8 MHz, DMSO-d_6_) *δ* 54.1, 88.9, 110.0, 111.3, 114.8, 115.6, 116.0, 116.2, 120.4, 122.2, 123.7, 128.5, 129.2–129.3 (m), 136.9, 141.4, 143.4, 150.5, 160.6–163.8 (m); HRMS for C_21_H_15_N_5_F [M^+^ + H] calcd. 356.13060, found 356.12971.

### 2.2. Leishmania Major Cell Isolation, Culture Conditions, and Assays

Promastigotes of *L. major* were isolated from a Saudi patient (February 2016) and maintained, at 26 °C, in Schneider’s Drosophila medium (Invitrogen, Carlsbad, CA, USA) containing 10% heat inactivated fetal bovine serum (FBS, Invitrogen, USA) and antibiotics in a tissue culture flask with weekly transfers. Promastigotes were cryopreserved in liquid nitrogen at concentrations of 3 × 10^6^ parasite/mL. Virulent *L. major* parasites were maintained by passing in female BALB/c mice by injecting hind footpads with 1 × 10^6^ stationary-phase promastigotes. *L. major* amastigotes were isolated from the mice after 8 weeks. Isolated amastigotes were converted to promastigotes by cultivation, at 26 °C, in Schneider’s medium supplemented with antibiotics and 10% FBS. Amastigote-derived promastigotes, which had undergone less than five in vitro passages, were used for infection. BALB/c mice (male and female individuals) were obtained from Pharmaceutical College, King Saud University, Kingdom of Saudi Arabia, and maintained in specific pathogen-free facilities. The handling of the laboratory animals followed the instructions and rules of the committee of research ethics, Deanship of Scientific Research, Qassim University, permission number 20-03-20.

*L. major* promastigotes from logarithmic-phase were cultured in phenol red-free RPMI 1640 medium (Invitrogen, USA) with 10% FBS and suspended on 96-wells plates to yield 10^6^ cells mL^−1^ (200 µL/well) after counting by a hemocytometer. Compounds were added to the wells, obtaining final concentrations of 50, 25, 12.5, 6.25, 3.13, 1.65, and 0.75 µg mL^−1^. Negative controls contained cultures with DMSO (1%) devoid of test compound and positive control wells contained cultures with decreasing concentrations of amphotericin B (50, 25, 12.5, 6.25, 3.13, 1.65, 0.75 µg mL^−1^) as active reference compound. After incubation, at 26 °C, for 72 h, the number of viable promastigotes was assessed by colorimetric method (tetrazolium salt colorimetric assay, MTT). The formed colored formazan was isolated and solubilized by addition of a detergent solution. The samples were analyzed by using an ELISA reader at 570 nm. EC_50_ values were calculated from three independent experiments [15].

For evaluation of the activity against amastigotes in macrophages, peritoneal macrophages were collected from female BALB/c mice (6–8 weeks of age) by aspiration. A total of 5 × 10^4^ cells/well were placed into 96-well plates containing phenol red-free RPMI 1640 medium with 10% FBS and were incubated to promote cell adhesion, at 37 °C, for 4 h in 5% CO_2_ atmosphere. Thereafter, the medium was discarded, and the cells were washed with phosphate-buffered saline (PBS). *L. major* promastigotes solution (200 µL at a ratio of 10 promastigotes:1 macrophage in RPMI 1640 medium with 10% FBS) was added to each well, and the plates were incubated for 24 h, at 37 °C, in humidified 5% CO_2_ atmosphere to enable macrophage infection and amastigote differentiation. The infected cells were washed three times with PBS to remove the free promastigotes and overlaid with fresh phenol red-free RPMI 1640 medium containing test compounds (50, 25, 12.5, 6.25, 3.13, 1.65, and 0.75 µg mL^−1^), whereupon the cells were incubated, at 37 °C, for 72 h in humidified 5% CO_2_ atmosphere. Cultures solely containing DMSO (1%) were used as negative controls, while wells containing cultures with decreasing concentrations of amphotericin B (reference compound, 50, 25, 12.5, 6.25, 3.13, 1.65, and 0.75 µg mL^−1^) were used as positive control. The percentage of infected macrophages was evaluated microscopically after the removal of the medium, washing, fixation, and Giemsa staining of the cells. Calculated EC_50_ values were obtained from three independent experiments [15].

### 2.3. Toxoplasma Gondii Cell Line, Culture Conditions, and Assay

Serial passages of cells of the Vero cell line (ATCC^®^ CCL81™, Manassas, VA, USA) were applied for the cultivation of *T. gondii* tachyzoites of the RH strain (a gift from Dr. Saeed El-Ashram, State Key Laboratory for Agrobiotechnology, China Agricultural University, Beijing, China). Vero cells were cultured by using complete RPMI 1640 medium with heat-inactivated 10% FBS in a humidified 5% CO_2_ atmosphere, at 37 °C. The 96-well plates (5 × 10^3^ cells/well in 200 µL RPMI 1640 medium) were used for the cultivation of the Vero cells and then incubated, at 37 °C, and 5% CO_2_ for one day, followed by removal of medium and washing the cells with PBS. Then, RPMI 1640 medium containing 2% FBS and *T. gondii* tachyzoites (RH strain) was given to the cells at a ratio of 5 (parasite):1 (Vero cells). After incubation, at 37 °C, and 5% CO_2_ for 5 h, cells were washed with PBS and then treated as described below.

Control: RPMI 1640 medium containing DMSO (1%)

Experimental: Medium + compounds (dissolved in DMSO) (50, 25, 12.5, 6.25, 3.13, 1.65, and 0.75 µg mL^−1^).

After incubation, at 37 °C, and 5% CO_2_ for 72 h, the cells were washed with PBS, fixed in 10% formalin and stained with 1% toluidine blue. Examination of the cells and determination of the infection index (number of cells infected from 200 cells tested) of *T. gondii* were carried out with an inverted photomicroscope. The following equation was used for the calculation of the inhibition in %:

Inhibition (%) = (I Control − I Experimental)/(I Control) × 100

where “I Control” refers to the infection index of untreated cells and “I Experimental” refers to the infection index of cells treated with test compounds.

Then, effects of test compounds on parasite growth were expressed as EC_50_ (effective concentration at 50%) values. EC_50_ values were obtained from three independent experiments [15,16].

### 2.4. In Vitro Cytotoxicity Assay

MTT assays were carried out for a cytotoxicity evaluation of the test compounds. Briefly, Vero cells were cultured in 96-well plates (5 × 10^3^ cells/well/200 µL) for 24 h in RPMI 1640 medium with 10% FBS and 5% CO_2_, at 37 °C. Cells were washed with PBS, followed by treatment with test compounds for 72 h at varying concentrations (50, 25, 12.5, 6.25, 3.13, 1.65, and 0.75 µg mL^−1^) in 10% FBS medium. Cells treated solely with medium in 2% FBS were used as a negative control. The supernatant was discarded, 50 µL RPMI 1640 medium containing 14 µL MTT (5 mg mL^−1^) was added, and the cells were incubated for 4 h. The supernatant was removed again, and 150 µL DMSO was added in order to dissolve the formed formazan. A FLUOstar OPTIMA spectrophotometer was applied for colorimetric analysis (λ = 540 nm). The cytotoxicity was expressed by IC_50_ values (concentration which caused a 50% reduction in viable cells). IC_50_ values were calculated from three independent experiments [15].

## 3. Results

**Scheme 1:**
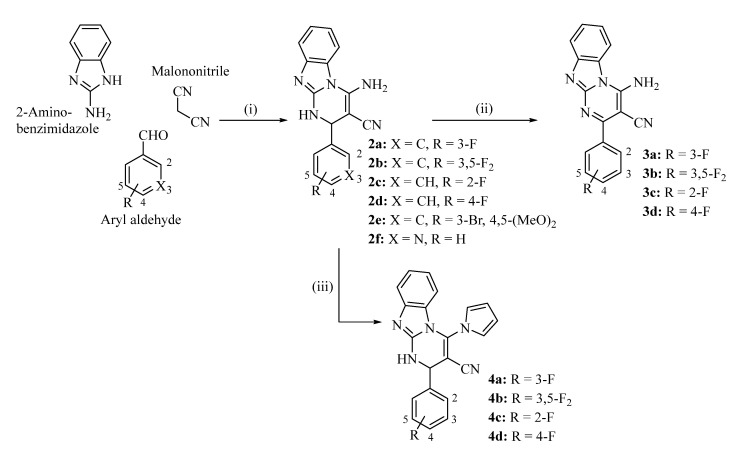
Reagents and conditions: (i) H_2_O, 80 °C, 24 h, 28–68%; (ii) *p*-chloranil, DMF, 120 °C, 5 min, then H_2_O/DMF, 120 °C, 3 min, 61–95%; (iii) 2,5-dimethoxytetrahydrofuran, AcOH, 90 °C, 3 h, 26–39%.

The 4-amino-3-cyano-2-aryl-1,2-dihydropyrimido[1,2-*a*]benzimidazoles **2a**–**f** were obtained from the three-component reaction of 2-aminobenzimidazole, malononitrile, and the corresponding aryl aldehyde under “green” conditions in hot H_2_O (Scheme 1). Compounds **2c** and **2d** were published before and were added to this compound series for comparison purposes in the following antiparasitic tests [13,14]. The fluorophenyl-substituted derivatives **2a**–**d** were used as starting materials for the synthesis of compounds **3a**–**d** and **4a**–**d**. Oxidation of **2a**–**d** with *p*-chloranil generated the pyrimido[1,2-*a*]benzimidazoles **3a**–**d**. Compounds **3a**–**c** are new, while **3d** was published before [13]. The reaction of **2a**–**d** with 2,5-dimethoxytetrahydrofuran led to the new pyrroles **4a**–**d**, which were prepared in order to evaluate the role of the 4-amino group of compounds **2a**–**d**.

Compounds **2a**–**f**, **3a**–**d**, and **4a**–**d** were initially tested for their activity against *T. gondii* tachyzoites (Table 1). Compound **3b** (IC_50_ = 2.8 µM) showed the highest activity against *T. gondii*, closely followed by **2a** (IC_50_ = 4.59 µM). **3b** was distinctly less toxic to the non-malignant Vero cells than **2a**, and exhibited a selectivity index (SI) of 8.12. In contrast, **2a** showed an activity against Vero cells similar to the activity against the *T. gondii* cells indicating no selectivity of this compound for *T. gondii*. The approved antiparasitic drug atovaquone (ATO) showed higher activity against *T. gondii* than **3b**, but ATO was also more toxic to Vero cells than **3b**.

The activity of compounds **2a**–**f**, **3a**–**d**, and **4a**–**d** against *L. major* promastigotes and amastigotes was also determined (Table 2). Again, compound **2a** performed well and showed excellent activities against *L. major* promastigotes and amastigotes (EC_50_ = 0.2–0.4 µM), which were higher than the activities of the approved antileishmanial drug amphotericin B (AmB). A considerable selectivity for *L. major* was observed for compound **2a** when compared with its activity against Vero cells (SI = 11.1 for promastigotes and 21.6 for amastigotes). Among the other compounds, only **2d**, **3b**, and **4c** showed moderate activities against the *L. major* promastigotes (IC_50_ = 13.7–15.2 µM).

In addition to Vero cells, the effects of selected test compounds on macrophages were investigated in order to obtain more information about the selectivity of the test compounds for the *L. major* amastigotes, which are growing within macrophages (Table 2). The most active antileishmanial compound **2a** was five times less active against macrophages (IC_50_ = 0.98 µM) than against *L. major* amastigotes (EC_50_ = 0.20 µM), indicating a moderate selectivity of this compound for amastigotes. Compounds **2b**–**d** and **3a**–**d** exhibited IC_50_ values of 20–24 µM, while compounds **4a**–**d** were distinctly less toxic, with IC_50_ values of 40–47 µM. The fluorine-free compounds **2e** and **2f** were least toxic to macrophages, with IC_50_ values of 64 µM and 86 µM, respectively. However, these compounds showed only low activity against *L. major* amastigotes, too.

**Table 2:** Effective concentrations EC_50_ (in µM for promastigotes and amastigotes of *Leishmania major* after 72 h) and inhibitory concentration IC_50_ (in µM for macrophages) of indicated test compounds. ^1^ Amphotericin B (AmB) was applied as a positive control.

Compd.	EC_50_ Promastigotes	EC_50_ Amastigotes	SI Vero/Promastigotes ^2^	SI Vero/Amastigotes ^2^	IC_50_ Macrophages
**2a**	0.39	0.20	11.1	21.6	0.98
**2b**	107.6	22.9	0.31	1.45	28.5
**2c**	49.8	24.9	0.37	0.74	31.4
**2d**	14.4	26.9	1.87	1.00	19.3
**2e**	77.9	75.5	0.73	0.76	63.8
**2f**	53.4	116.5	1.98	0.91	86.0
**3a**	35.6	28.0	1.08	1.37	22.4
**3b**	13.7	29.0	1.66	0.78	24.3
**3c**	32.0	27.0	0.80	0.95	21.1
**3d**	36.9	26.1	1.18	1.67	20.1
**4a**	20.3	50.9	1.72	0.69	41.4
**4b**	28.9	46.1	1.12	0.70	39.9
**4c**	15.2	49.2	1.55	0.48	43.9
**4d**	32.1	51.5	1.29	0.80	47.3
**AmB**	0.83	0.47	9.6	16.4	-

^1^ Means of *n* = 3, SD ± 15%. ^2^ Selectivity index (IC_50_ from Table 1/EC_50_).

## 4. Discussion

The test compounds used in this study were obtained in low-to-moderate yields. However, the synthesis of the 1,2-dihydropyrimido[1,2-*a*]benzimidazoles **2a**–**f** was accomplished by using one-pot “green” conditions. “Green chemistry” is of growing importance as a sustainable way to prepare drug candidates for the treatment of cancer and infectious diseases [13,17,18]. Compounds **2a**–**f** and **4a**–**d** were obtained and tested as racemic mixtures, and an increase in activity might be achieved by separation of the enantiomers of the active derivatives followed by testing against *T. gondii* and *L. major* parasites. In addition, chiral synthetic procedures by using chiral catalysts or additives might be applied to obtain enantiopure test compounds for biological testing.

In terms of antiparasitic activities, the new 4-amino-3-cyano-2-(3,5-difluorophenyl)-pyrimido[1,2-*a*]benzimidazole **3b** exhibited a high activity against and distinct selectivity for *T. gondii* cells, while its activity against *L. major* parasites, Vero cells, and macrophages was considerably lower. The reasons for this selectivity for *T. gondii* remain to be elucidated and are likely based on differences in the biology of *T. gondii* cells and *L. major*/Vero/macrophage cells. Based on its high selectivity and reduced toxicity when compared with atovaquone, **3b** might be considered for advanced biological testing using *T. gondii* parasite models in vitro and in vivo.

In contrast, very promising results were obtained from the evaluation of the new 4-amino-3-cyano-2-(3-fluorophenyl)-1,2-dihydropyrimido[1,2-*a*]benzimidazole **2a** in *L. major* parasites. Both excellent activities against and considerable selectivities for *L. major* promastigotes and amastigotes were discovered. It is remarkable that **2a** was more active than amphotericin B, and it showed slightly higher activity against *L. major* amastigotes than against promastigotes of this parasite. The high activity of **2a** against *L. major* amastigotes is of special interest since many clinically approved antileishmanial drugs also perform well against intracellular/intra-macrophage amastigotes [19,20]. Assays with amastigotes cover host cell mechanisms and, thus, provide more information about the properties of test compounds within human cells and about the potential of an active test compound to become an antileishmanial drug in the future [21,22]. Thus, **2a** has the potential to proceed to advanced stages of antileishmanial testing including in vivo experiments with suitable CL animal models. More detailed research about the mechanisms of action is warranted in order to identify the reason for the promising antileishmanial effects of **2a**. The combination of compound **2a** with approved antileishmanial drugs would also be useful in order to optimize the efficacy, and to reduce the minimal effective dosage and possible side-effects in future animal studies.

Structure–activity relationships were difficult to identify because only **2a** and **3b** showed reasonable antiparasitic activities. Slight modifications of **2a** (oxidation to pyrimido[1,2-*a*]benzimidazole **3a**, and modification of the 4-amino group to pyrrole **4a**) quickly led to distinctly reduced activities. Similarly, the reduced 1,2-dihydropyrimido[1,2-*a*]benzimidazole **2b** and pyrrole **4b** exhibited lower activities than their close analog **3b**. However, the fluoro-substitution pattern is critical too, and 3-fluorophenyl and 3,5-difluorophenyl compounds seem to be preferred in terms of activity. The underlying parasite-specific reasons for these differences in activity remain to be elucidated. However, 3,5-difluorophenyl-substituted pyrans were found to be especially active against cancer cells based on ROS formation and interference with microtubule dynamics [23]. In contrast, the 3-bromo-4,5-dimethoxyphenyl motif of compound **2d** exerted no antiparasitic activity, despite the well-documented apoptosis-inducing antitumor activities of this structural motif [24].

## 5. Conclusions

Two promising drug candidates against *L. major* and *T. gondii* were identified in this study. In particular, the high antileishmanial activity of **2a** warrants further investigation in terms of possible mechanisms of action and its activity against other *Leishmania* species as well as Kinetoplastea parasites such as *Trypanosoma*.

## Figures and Tables

**Table 1:** Inhibitory concentrations IC_50_ (in µM, after 72 h) of test compounds in Vero cells (African green monkey kidney epithelial cells), and effective concentrations EC_50_ (in µM, after 72 h) in *Toxoplasma gondii*. ^1^ Amphotericin B (AmB) and atovaquone (ATO) served as positive control drugs.

Compd.	EC_50_ (*T. gondii*)	IC_50_ (Vero)	SI (Vero/*T. gondii*) ^2^
**2a**	4.59	4.32	0.94
**2b**	43.6	33.1	0.76
**2c**	26.9	18.3	0.68
**2d**	27.8	26.9	0.97
**2e**	28.6	57.2	2.00
**2f**	60.0	105.5	1.75
**3a**	35.9	38.3	1.07
**3b**	2.80	22.7	8.12
**3c**	20.8	25.7	1.24
**3d**	41.9	43.5	1.04
**4a**	33.2	34.9	1.05
**4b**	18.2	32.4	1.78
**4c**	24.8	23.6	0.95
**4d**	34.9	41.4	1.19
**AmB**	-	7.7	-
**ATO**	0.07	9.5	136

^1^ Means of *n* = 3, SD ± 15%. ^2^ Selectivity index (IC_50_/EC_50_).

## Data Availability

Data supporting the reported results can be obtained from the authors upon request.

## Supplementary Materials

The following supporting information can be downloaded at: https://www.mdpi.com/article/10.3390/biomedicines11010219/s1, Figures S1–S33: syntheses of the known compounds **2c**, **2d**, and **3d**, ^1^H and ^13^C NMR spectra, and HRMS of new compounds.
