# Supplementary material for: Antiparasitic Activity of Fluorophenyl-Substituted Pyrimido[1,2-a]benzimidazoles
Source: Biomedicines. 2023 Jan 14;11(1):219. doi: 10.3390/biomedicines11010219 (PMC9855371; doi:10.3390/biomedicines11010219)
Supplement: Supplementary file 1 [file biomedicines-11-00219-s001.zip › biomedicines-2144591-supplementary.pdf]

Supplementary Materials

Antiparasitic activity of fluorophenyl-substituted pyrimido[1,2-*a*]benzimidazoles

Ibrahim S. Al Nasr [1,2], Waleed S. Koko [2], Tariq A. Khan [3], Rainer Schobert [4] and Bernhard Biersack [4,*]

[1] Department of Biology, College of Science and Arts, Qassim University, Unaizah 51911, Saudi Arabia; insar@qu.edu.sa
[2] Department of Science Laboratories, College of Science and Arts, Qassim University, King Abdelaziz Road, Ar Rass 51921, Saudi Arabia; insar@qu.edu.sa, wasyko2002@yahoo.com
[3] College of Applied Health Sciences, Qassim University, Ar Rass 51921, Saudi Arabia; sirtariqayub@gmail.com
[4] Organic Chemistry Laboratory, University Bayreuth, Universitätsstrasse 30, 95440 Bayreuth, Germany; rainer.schobert@uni-bayreuth.de, bernhard.biersack@uni-bayreuth.de

***** Correspondence: bernhard.biersack@yahoo.com, bernhard.biersack@uni-bayreuth.de; Tel.: 0049-921-552673

Synthesis of known compounds **2c**, **2d**, and **3d**

4-Amino-2-(2-fluorophenyl)-1,2-dihydropyrimido[1,2-*a*]benzimidazole-3-carbonitrile (**2c**)

Malononitrile (33 mg, 0.5 mmol), 2-aminobenzimidazole (66 mg, 0.5 mmol), and 2-fluorobenzaldehyde (62 mg, 0.5 mmol) were suspended in H_2O (5 mL) and the reaction mixture was stirred at 80 °C for 24 h. The formed precipitate was collected, washed with water, a small amount of ethanol and diethyl ether and dried in vacuum. Yield: 83 mg (0.27 mmol, 54%); ^{1}H NMR (300 MHz, DMSO-d_6) δ 5.45 (1 H, s), 6.85 (2 H, s), 7.0-7.1 (1 H, m), 7.1-7.3 (5 H, m), 7.3-7.4 (1 H, m), 7.64 (1 H, d, J = 8.0 Hz), 8.51 (1 H, s).

4-Amino-2-(4-fluorophenyl)-1,2-dihydropyrimido[1,2-*a*]benzimidazole-3-carbonitrile (**2d**)

Malononitrile (33 mg, 0.5 mmol), 2-aminobenzimidazole (66 mg, 0.5 mmol), and 4-fluorobenzaldehyde (62 mg, 0.5 mmol) were suspended in H_2O (5 mL) and the reaction mixture was stirred at 80 °C for 24 h. The formed precipitate was collected, washed with water, a small amount of ethanol and diethyl ether and dried in vacuum. Yield: 79 mg (0.26 mmol, 52%); ^{1}H NMR (500 MHz, DMSO-d_6) δ 5.25 (1 H, s), 6.84 (2 H, s), 7.0-7.1 (1 H, m), 7.1-7.3 (4 H, m), 7.3-7.4 (1 H, m), 7.63 (1 H, d, J = 8.0 Hz), 8.56 (1 H, s).

4-Amino-2-(4-fluorophenyl)pyrimido[1,2-*a*]benzimidazole-3-carbonitrile (**3d**)

2d (25 mg, 0.082 mmol) was dissolved in hot DMF (1 mL) and *p*-chloranil (21 mg, 0.085 mmol) was added. The reaction mixture was stirred at 120 °C for 5 min. H_2O (0.1 mL) was added and the reaction mixture was stirred at 120 °C for 3 min. After cooling to room temperature, the formed precipitate was collected and washed with ethanol. Yield: 17 mg (0.056 mmol, 68%); ^{1}H NMR (500 MHz, DMSO-d_6) δ 7.3-7.5 (3 H, m), 7.5-7.6 (1 H, m), 7.7-7.8 (1 H, m), 7.9-8.0 (2 H, m), 8.5-8.6 (1 H, m), 8.8-8.9 (2 H, br s).

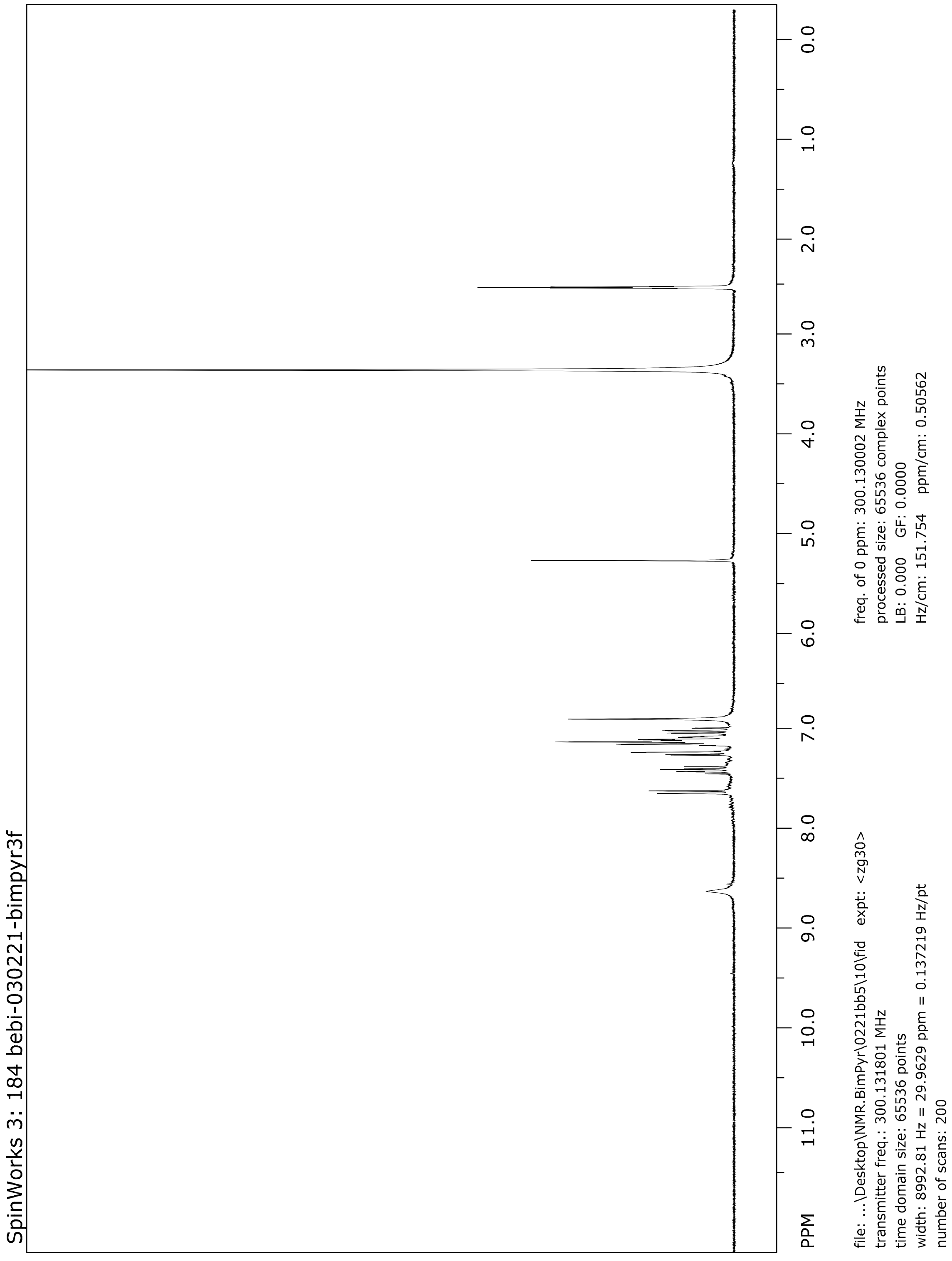

Figure S1. ^{1}H NMR spectrum of **2a**

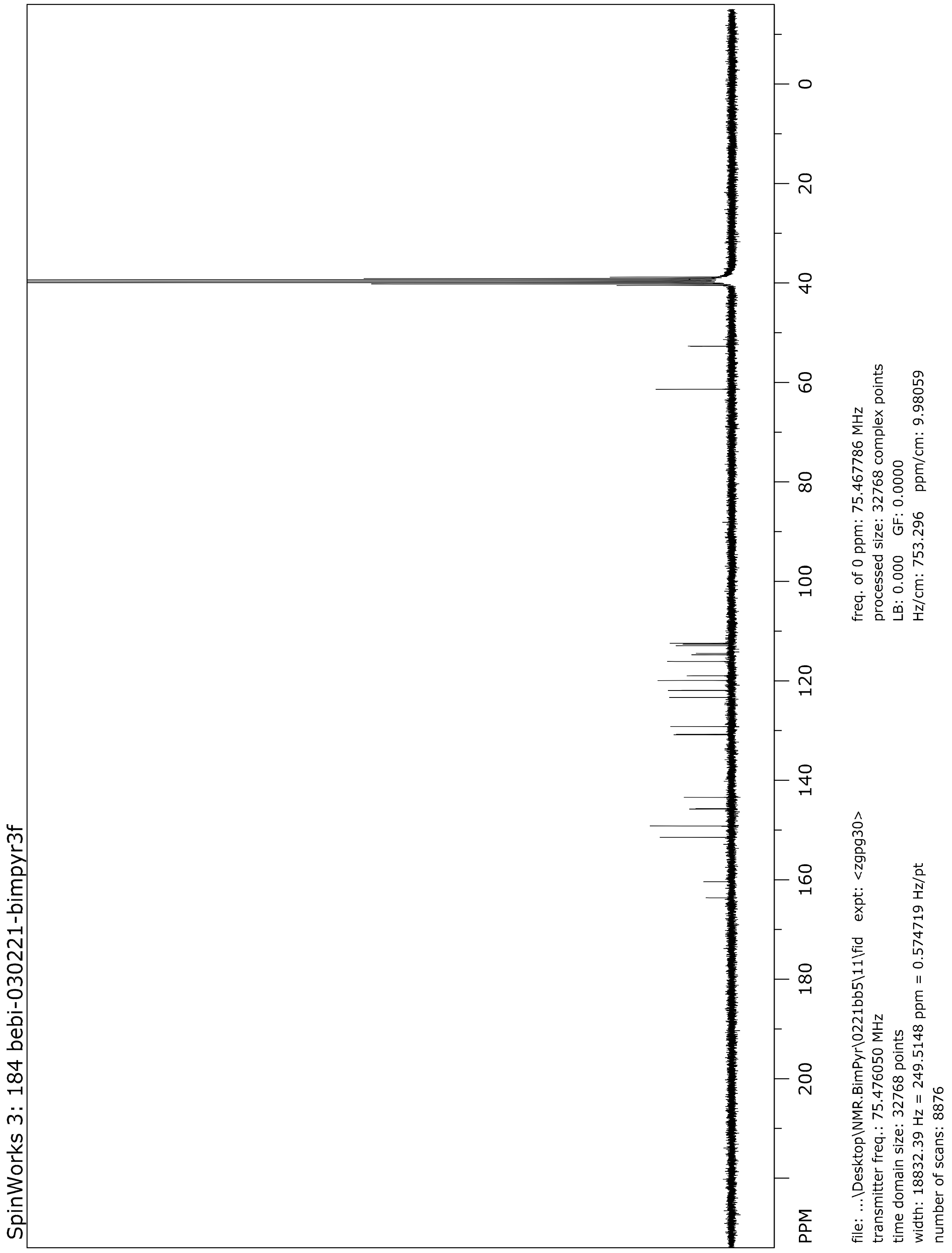

Figure S2. ^{13}C NMR spectrum of **2a**

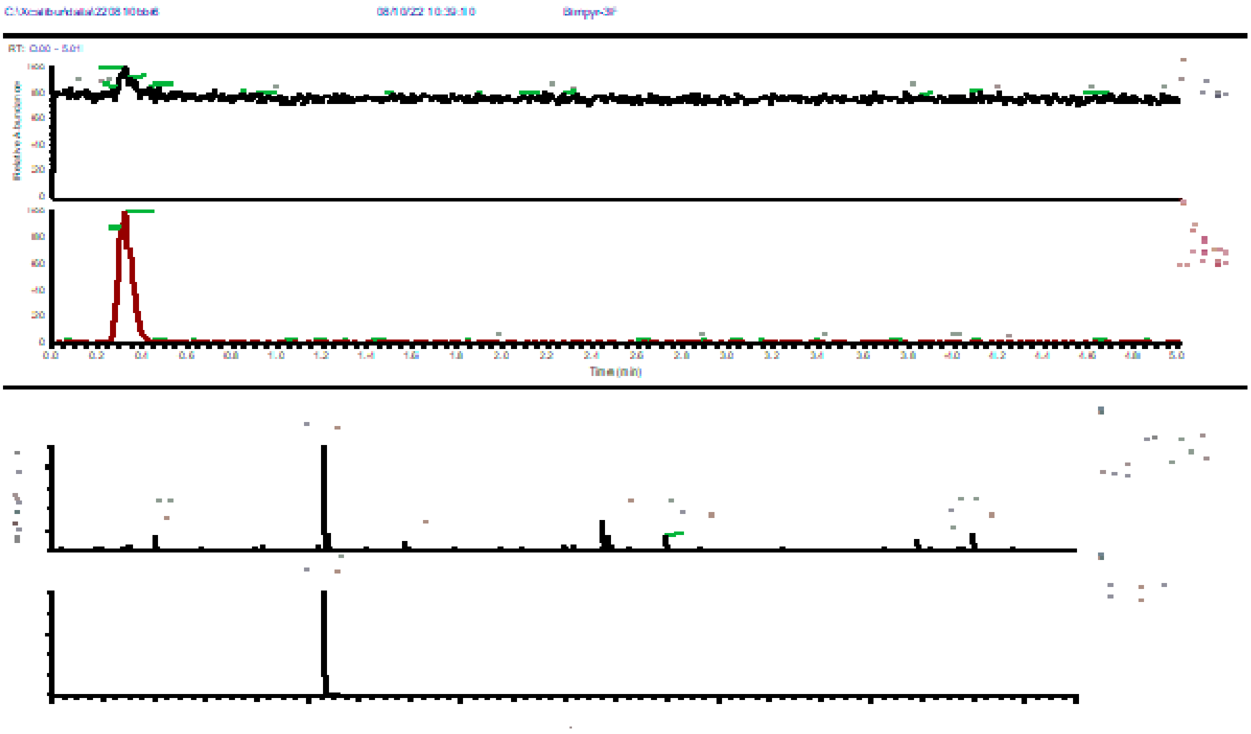

Figure S3. HRMS of **2a**

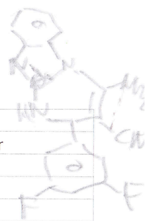

This report was created by ACD/N[illegible]R Processor Academic Edition. For more info[illegible]ation go to www.acdlabs.com/nmrproc/

bebi-080917-bp35df-1H

Acquisition Time (sec)	3.2768	*Comment*	Group AK Schobert bebi-080917-bp35df	*Date*	14 Sep 2017 04:01:36
Date Stamp	14 Sep 2017 04:01:36	*File Name*	\\132.180.48.106\AK_Schobert_NMR\Rehm_Tobias\bebi-080917-bp35df\10\PDATA\1\1r		
Frequency (MHz)	500.13	*Nucleus*	1H	*Number of Transients*	16
Origin	spect				
Original Points Count	32768	*Owner*	nmrsu	*Points Count*	65536
Pulse Sequence	zg30				
Receiver Gain	32.00	*SW(cyclical) (Hz)*	10000.00	*Solvent*	DMSO-d6
Spectrum Offset (Hz)	3083.8975				
Spectrum Type	STANDARD	*Sweep Width (Hz)*	9999.85	*Temperature (degree C)*	20.000

^{1}H NMR (500 MHz, DMSO-d_6) δ ppm 5.34 (1 H, s), 6.93 - 7.05 (5 H, m), 7.13 (1 H, t, J=7.6 Hz), 7.19 (1 H, tt, J=9.2, 2.3 Hz), 7.25 (1 H, d, J=7.8 Hz), 7.64 (1 H, d, J=8.1 Hz), 8.68 (1 H, br. s.)

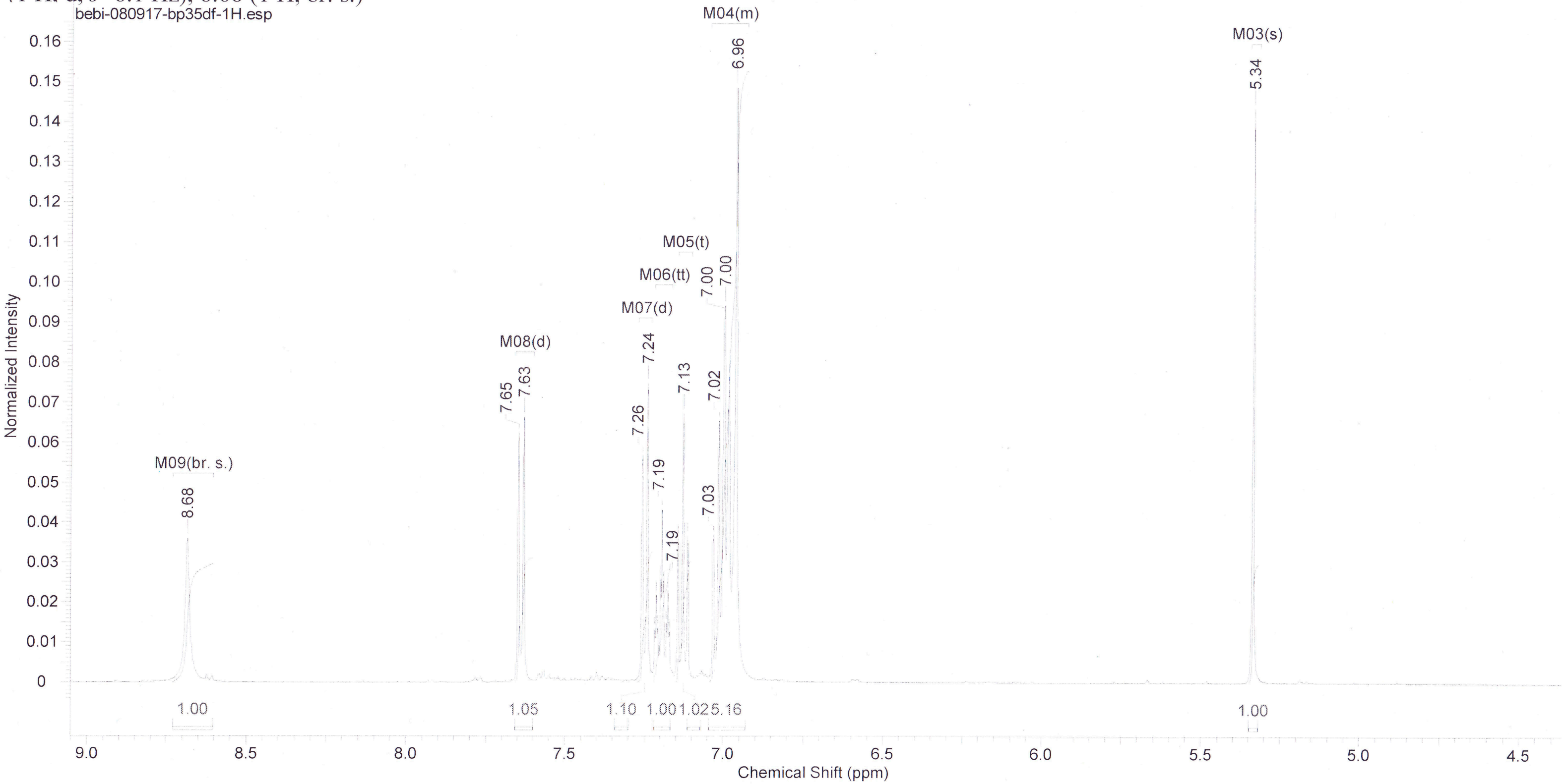

Figure S4. ^{1}H NMR spectrum of **2b**

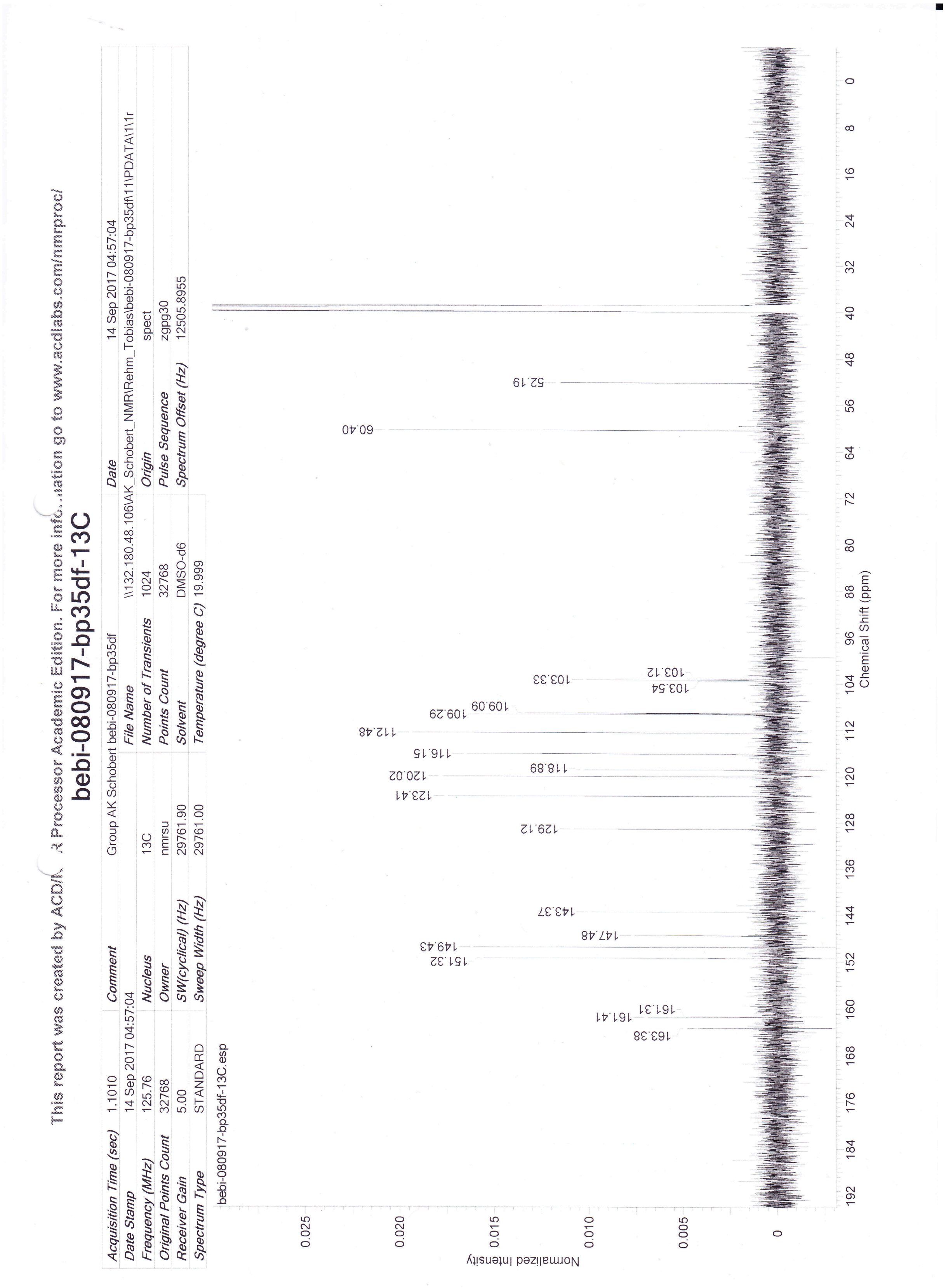

Figure S5. ^{13}C NMR spectrum of **2b**

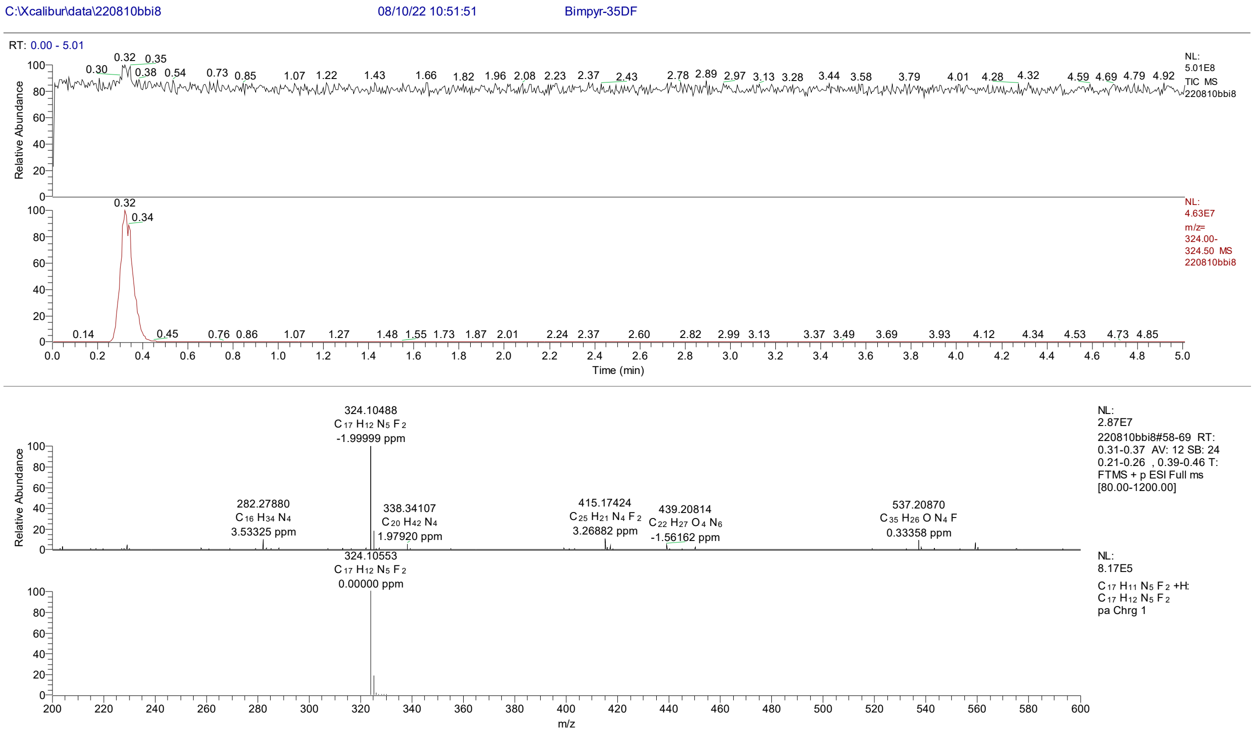

Figure S6. HRMS of **2b**

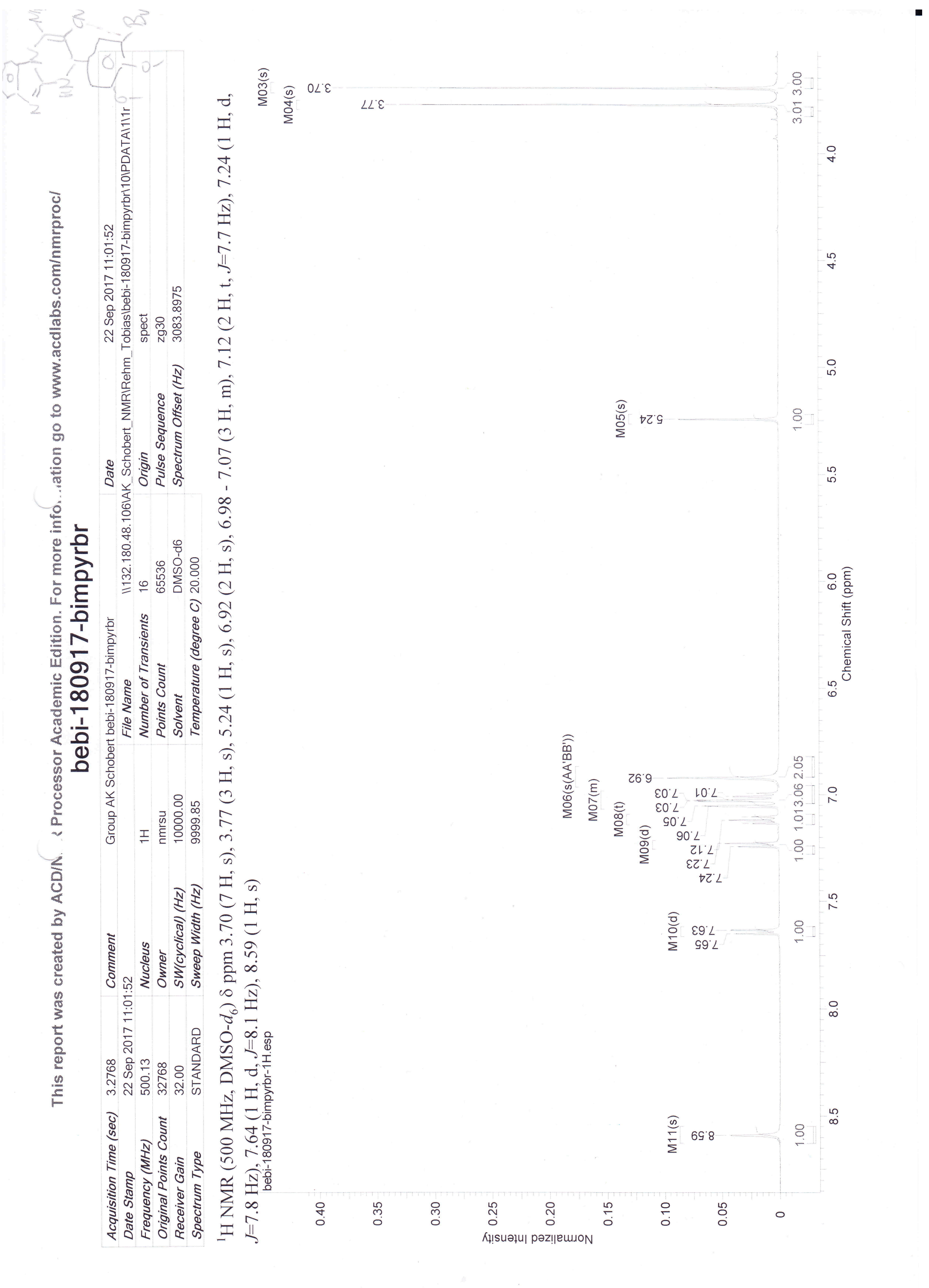

Figure S7. ¹H NMR spectrum of **2e**

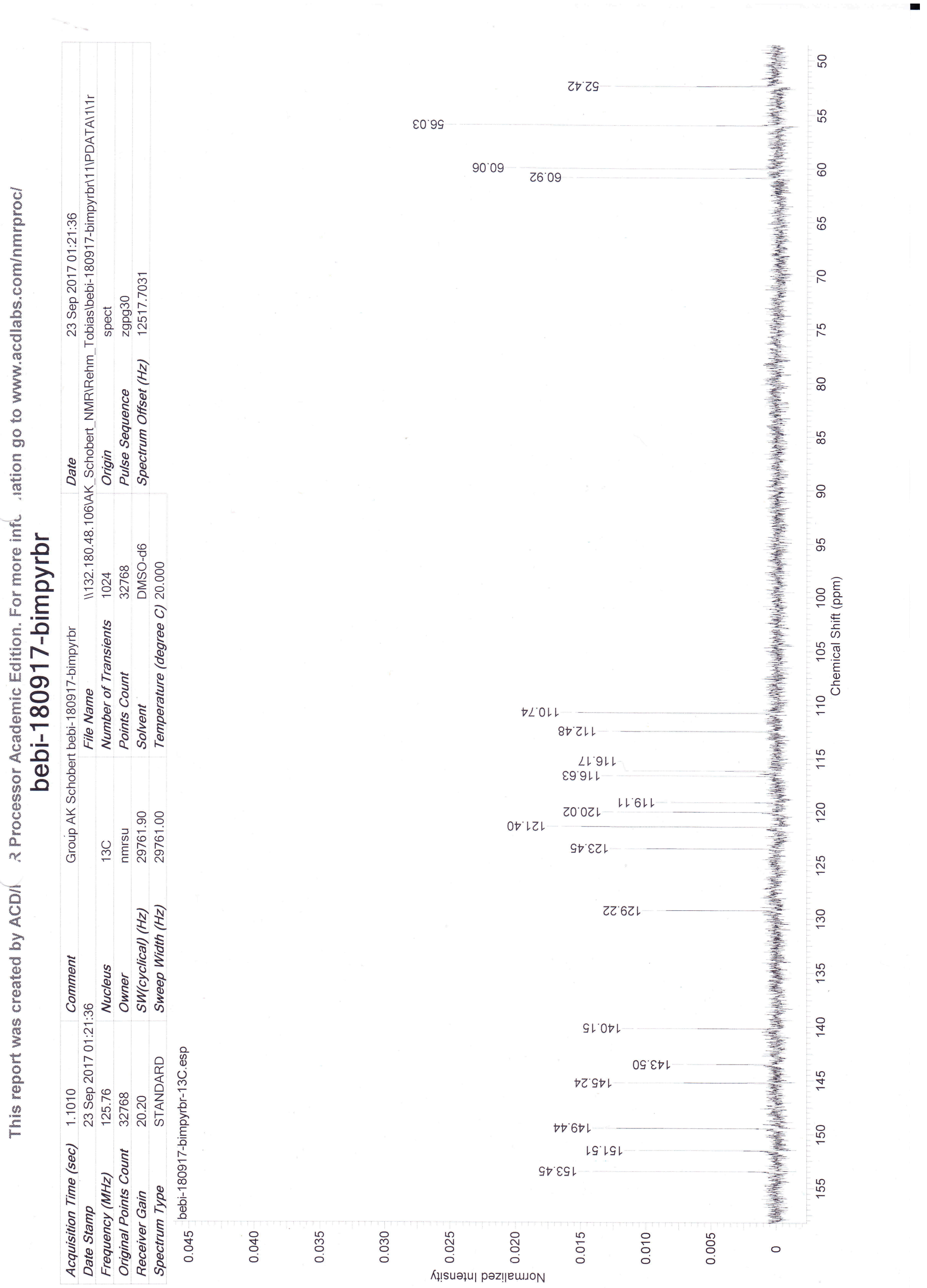

Figure S8. ^{13}C NMR spectrum of **2e**

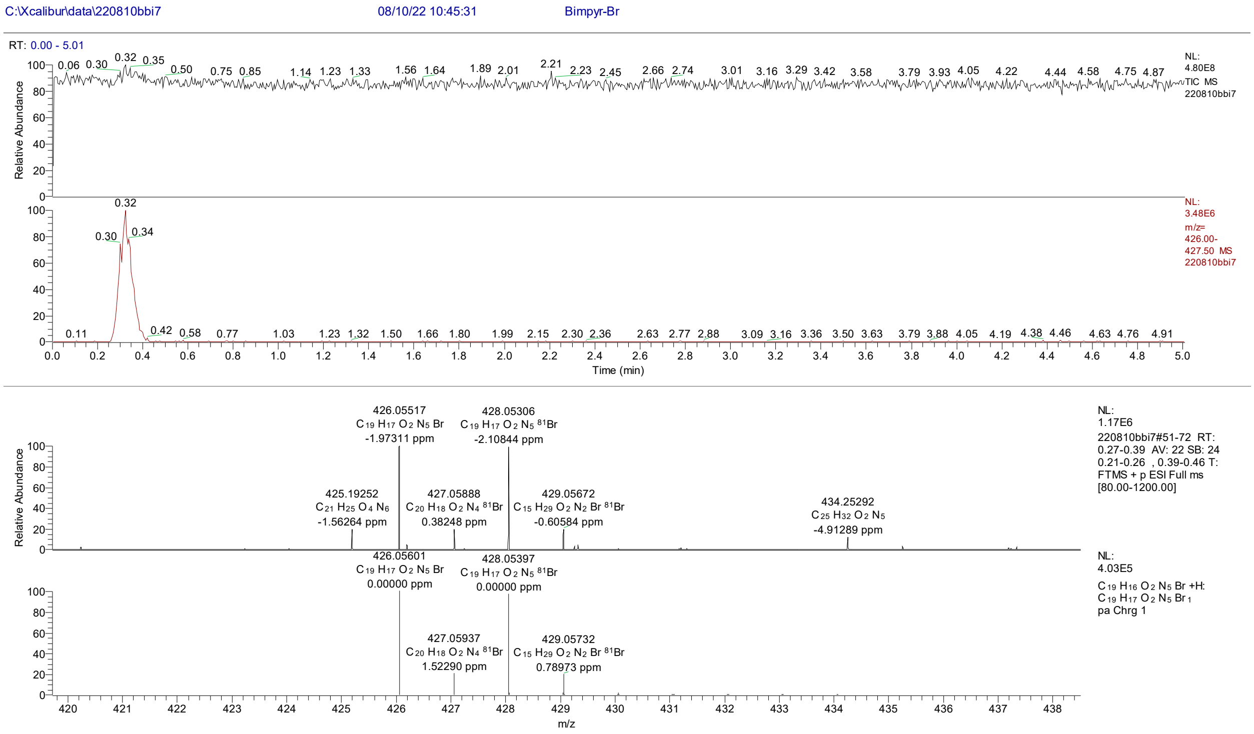

Figure S9. HRMS of **2e**

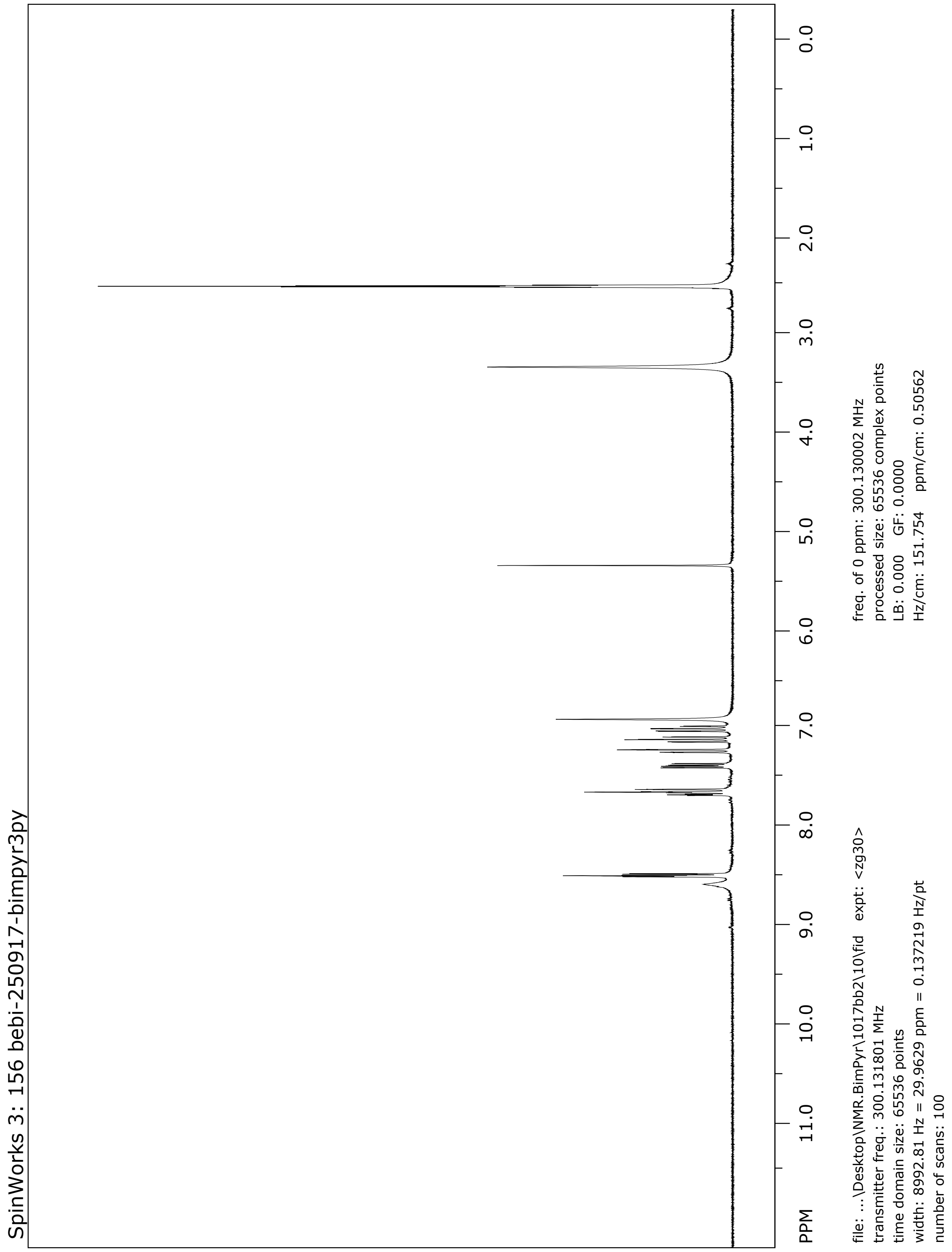

Figure S10. ^{1}H NMR spectrum of **2f**

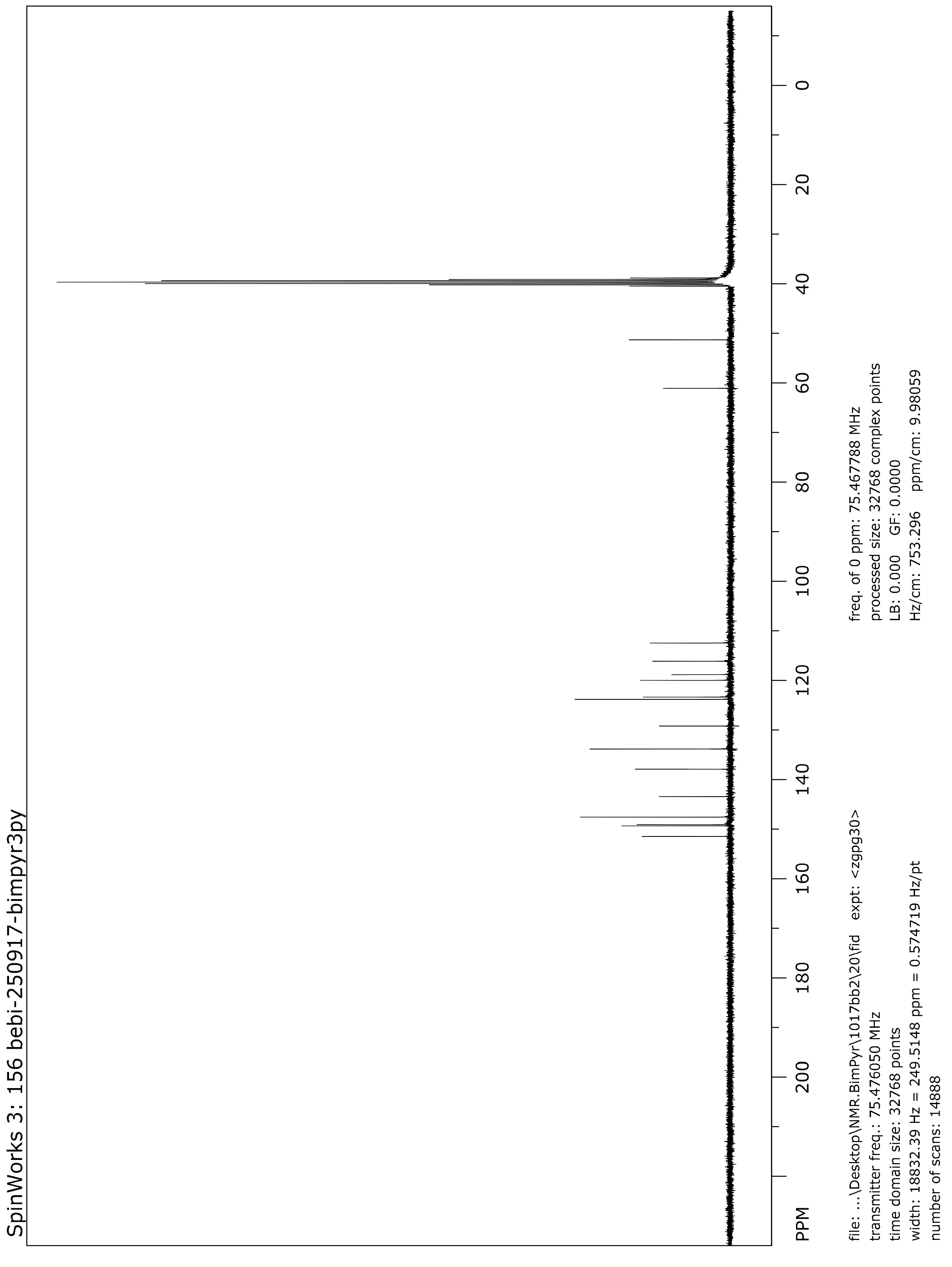

Figure S11. ^{13}C NMR spectrum of **2f**

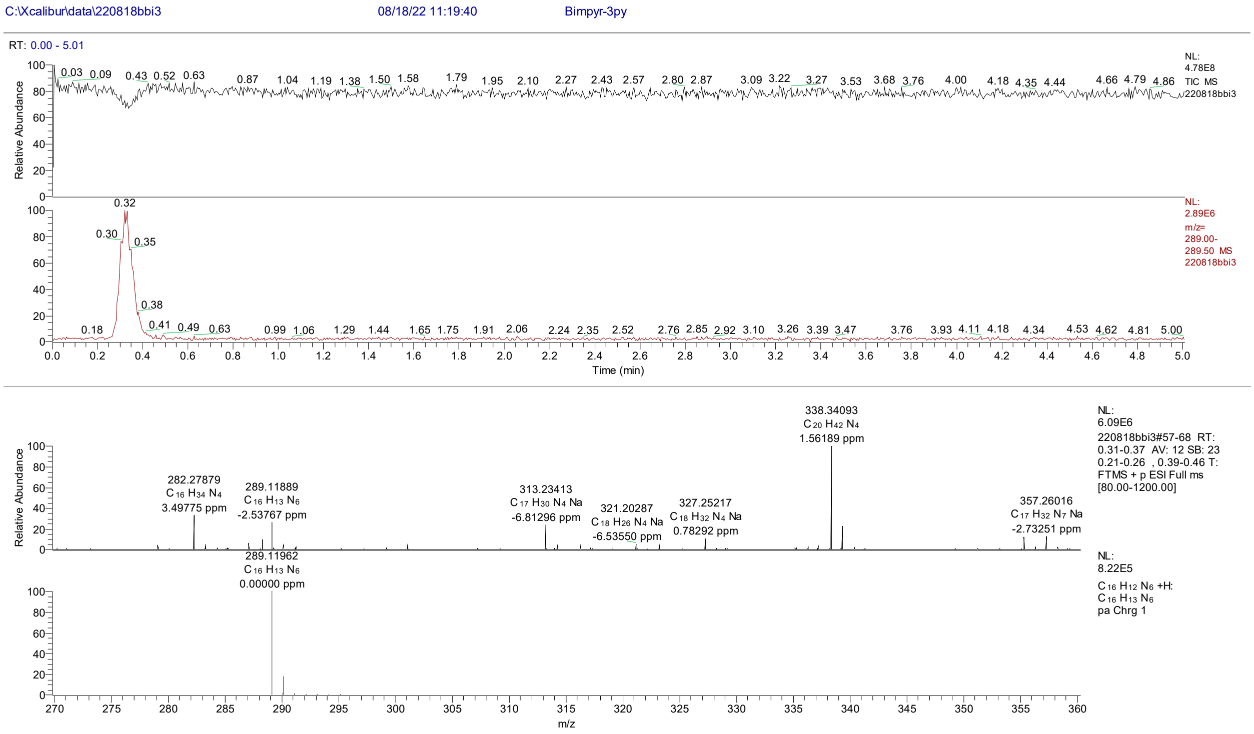

Figure S12. HRMS of **2f**

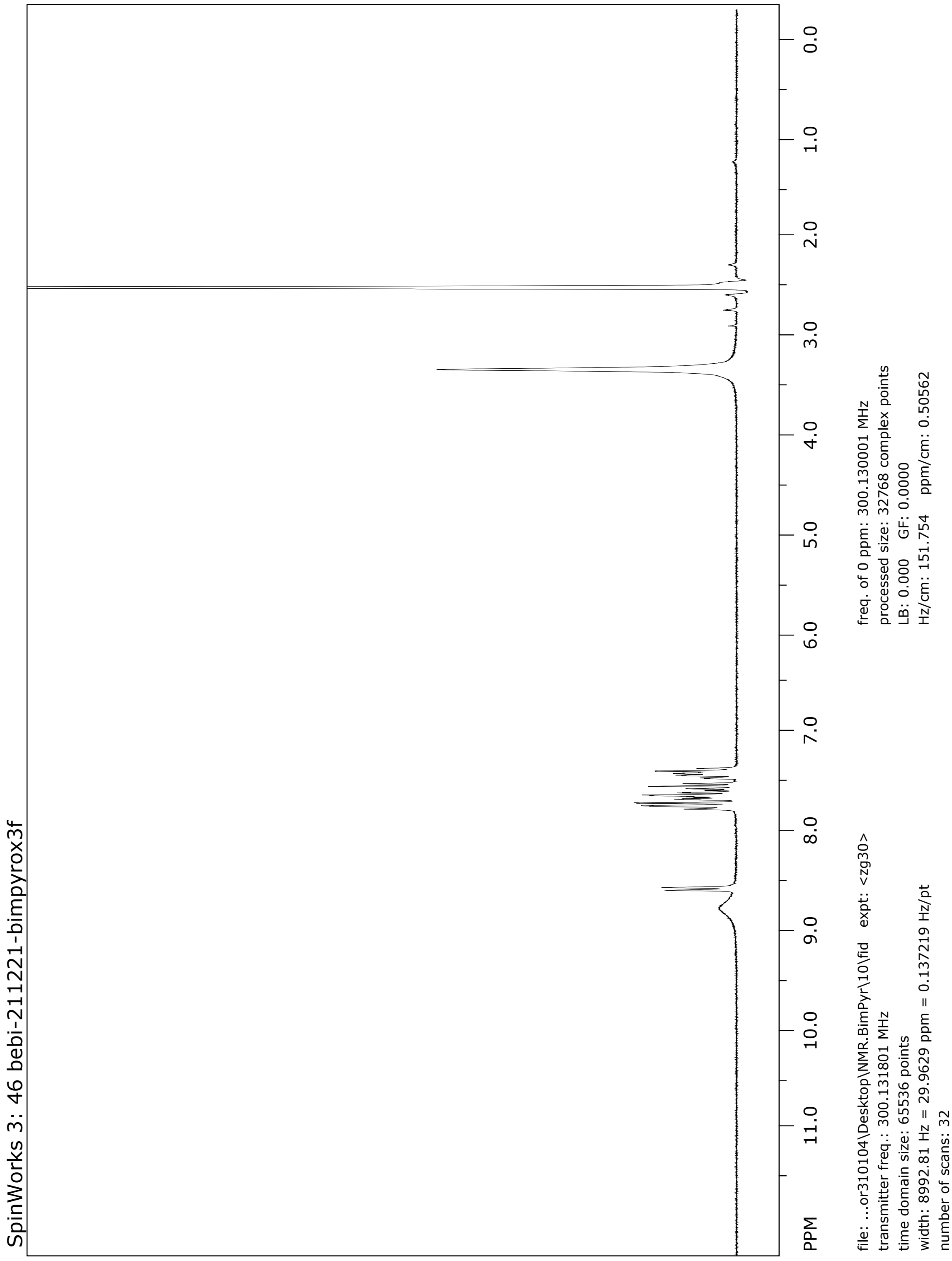

Figure S13. ^{1}H NMR spectrum of **3a**

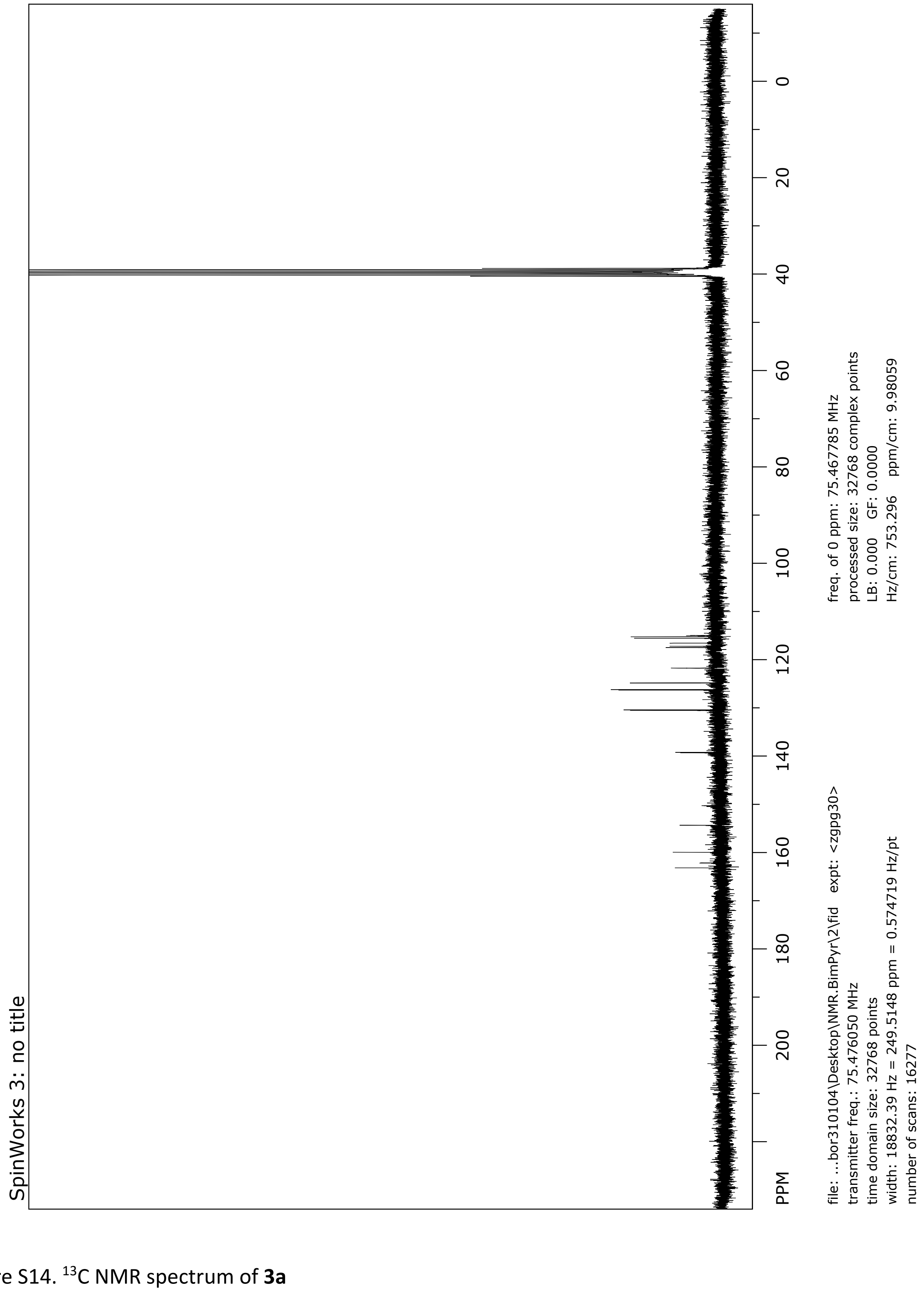

Figure S14. ^{13}C NMR spectrum of **3a**

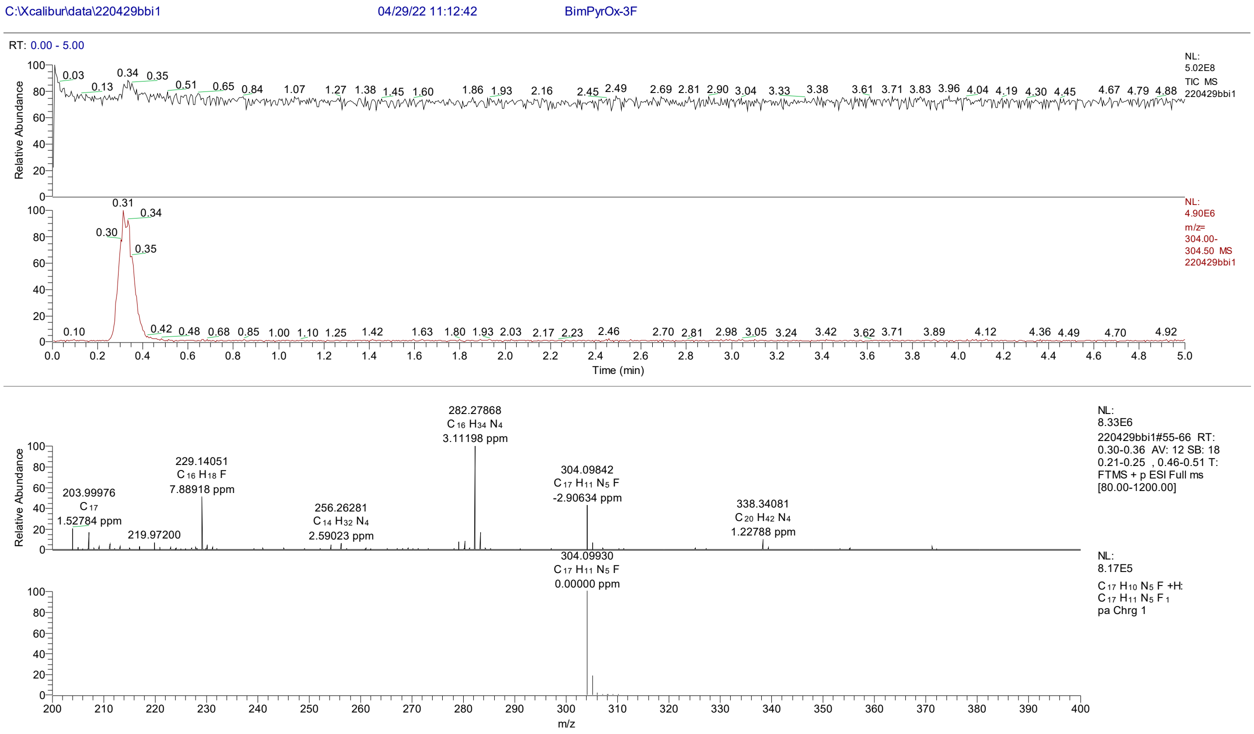

Figure S15. HRMS of **3a**

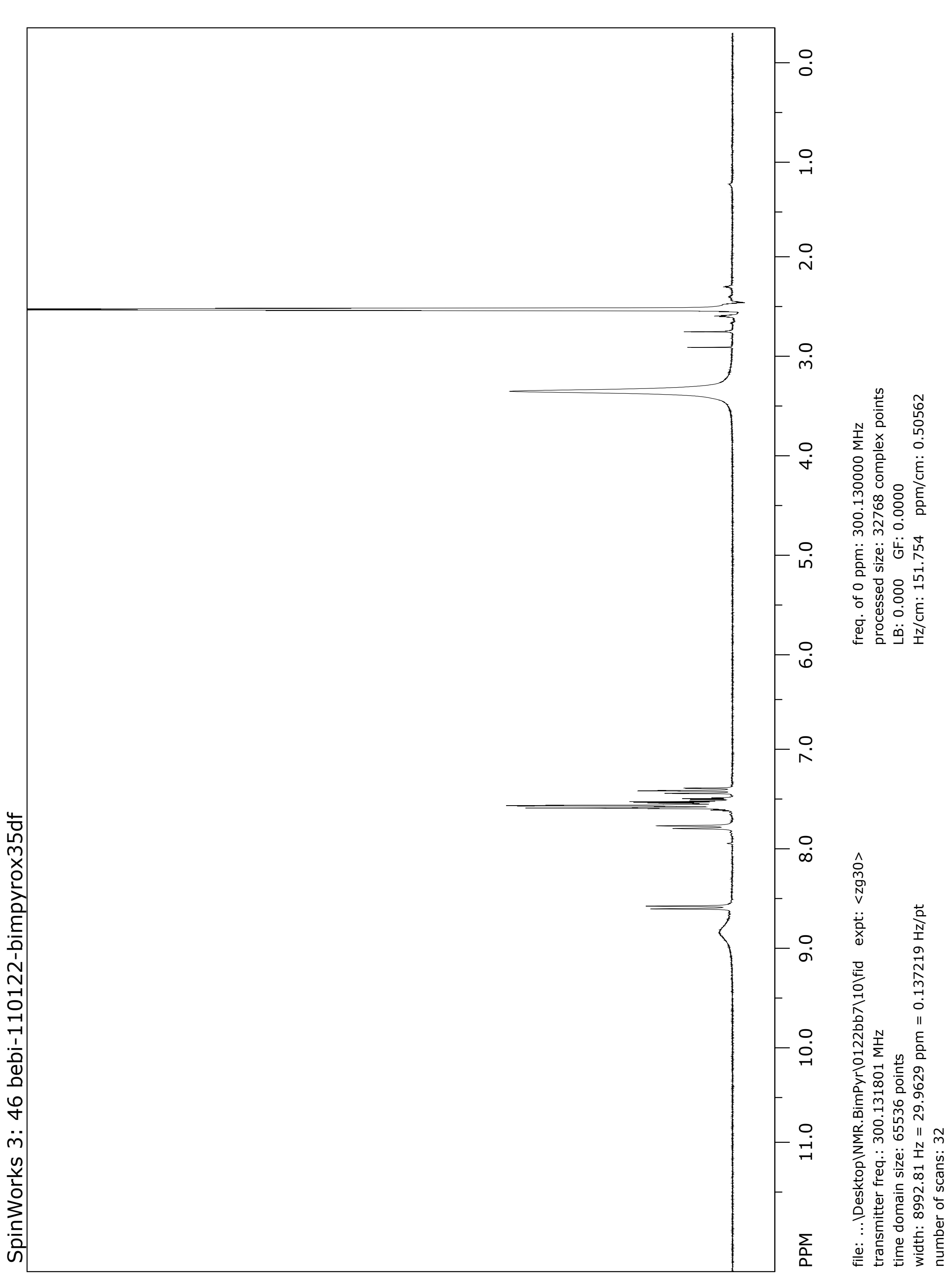

Figure S16. ^{1}H NMR spectrum of **3b**

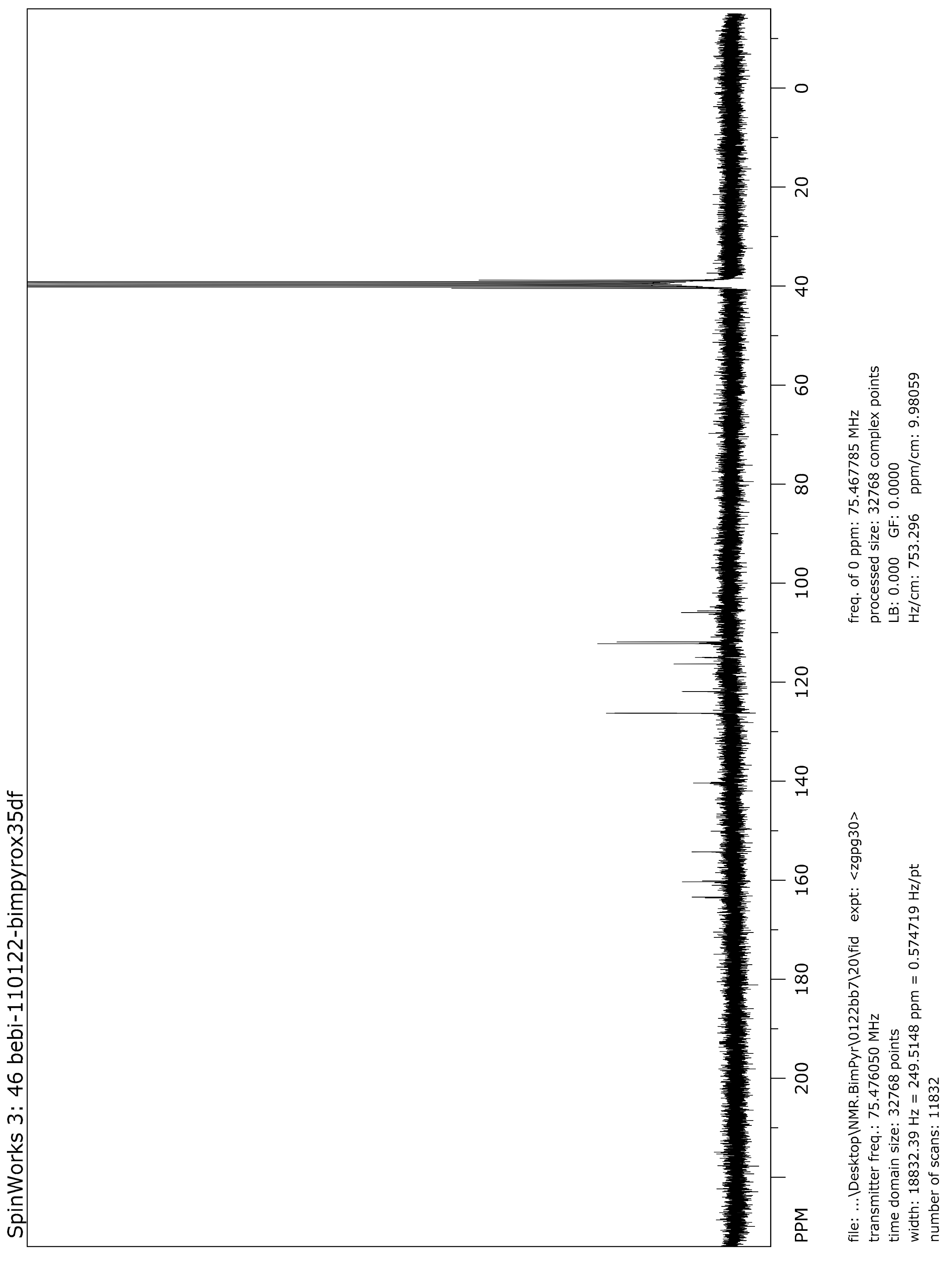

Figure S17. ^{13}C NMR spectrum of **3b**

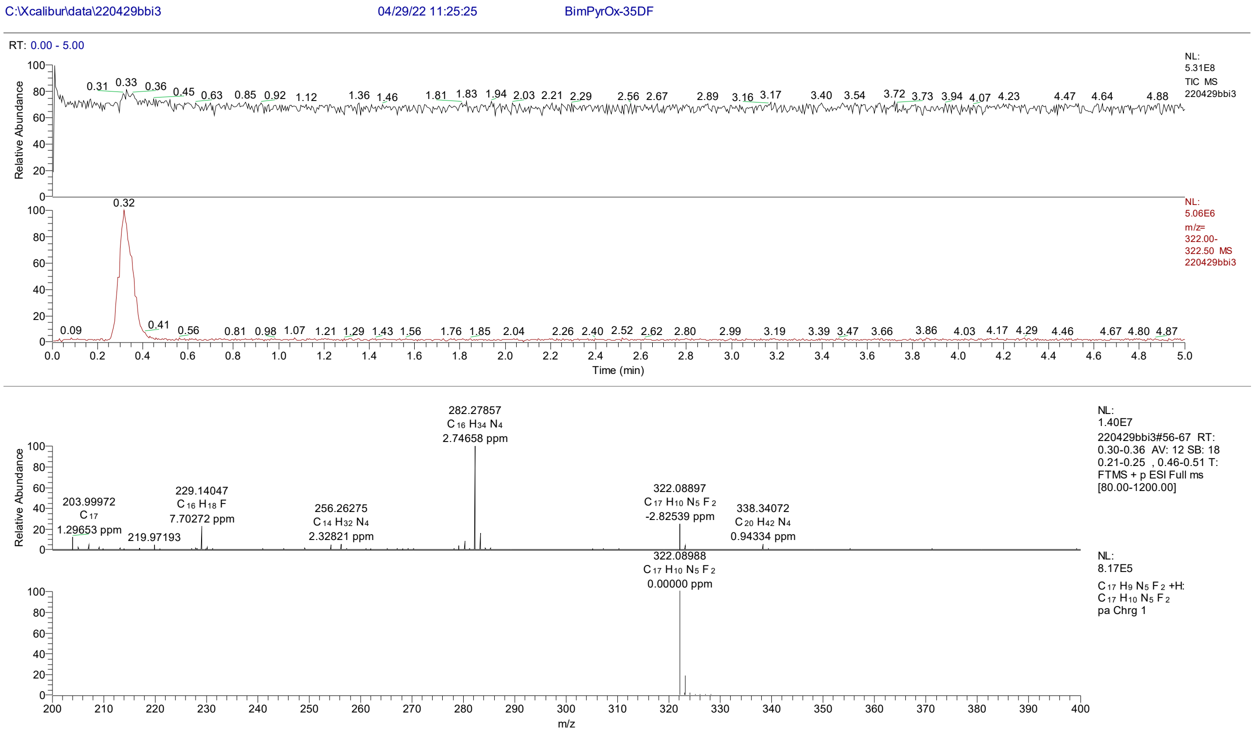

Figure S18. HRMS of **3b**

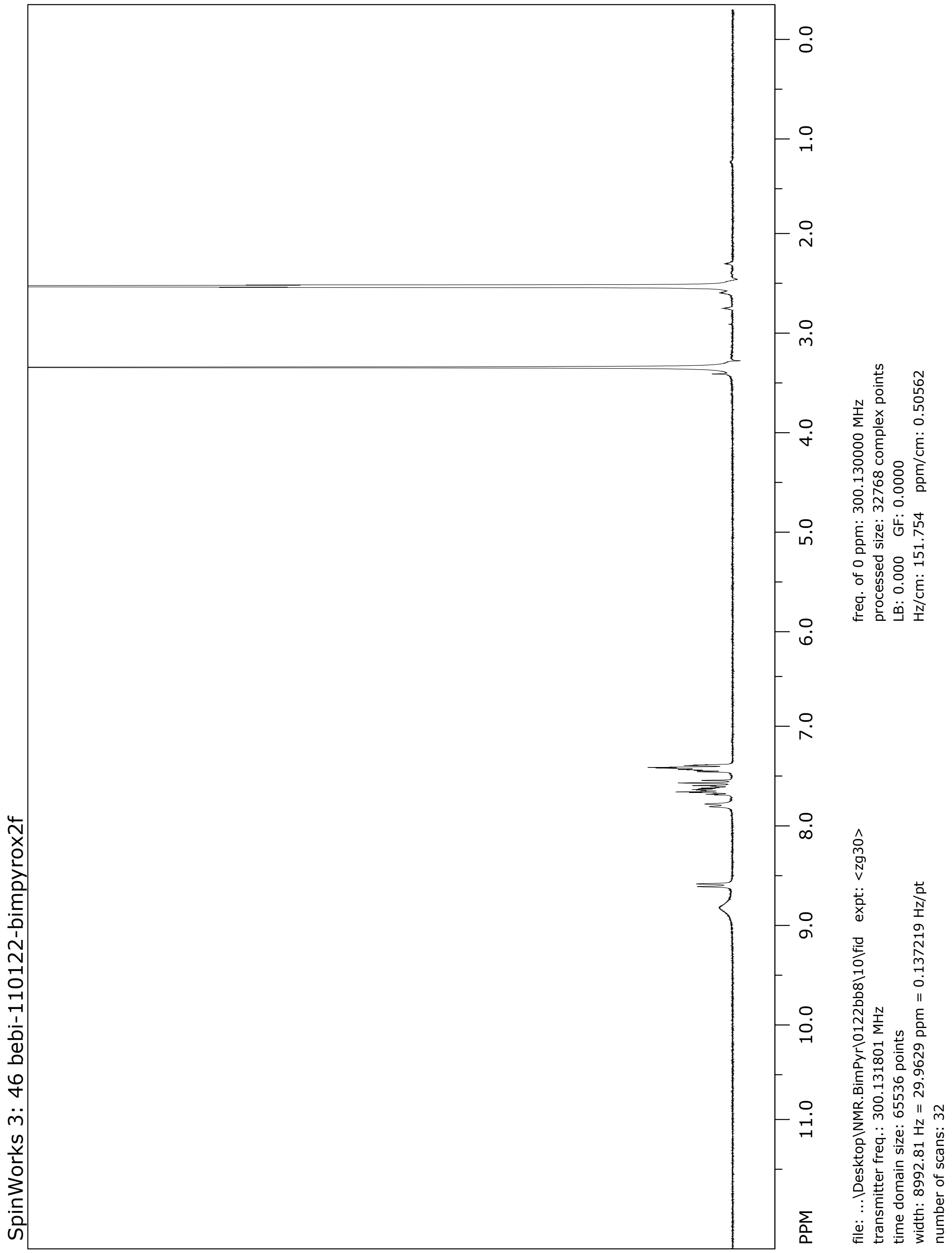

Figure S19. ^{1}H NMR spectrum of **3c**

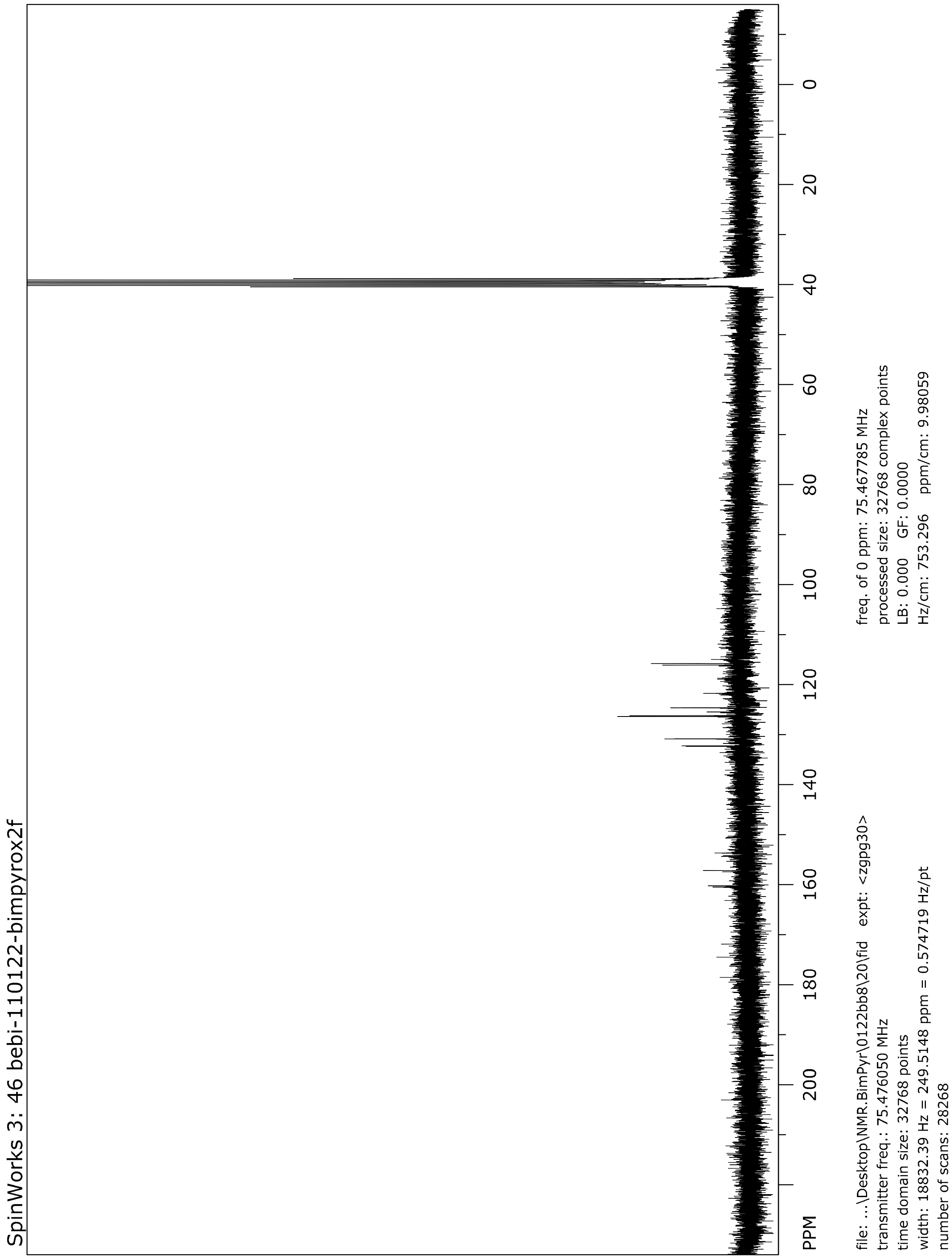

Figure S20. ^{13}C NMR spectrum of **3c**

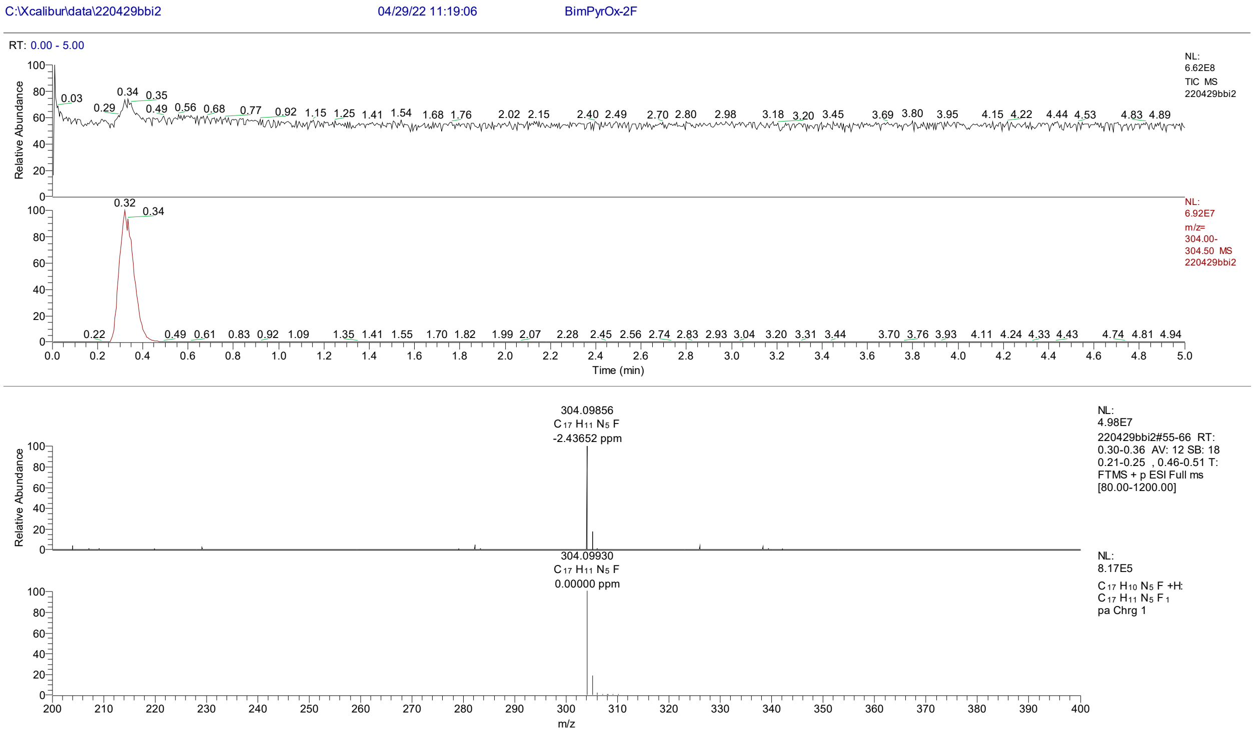

Figure S21. HRMS of **3c**

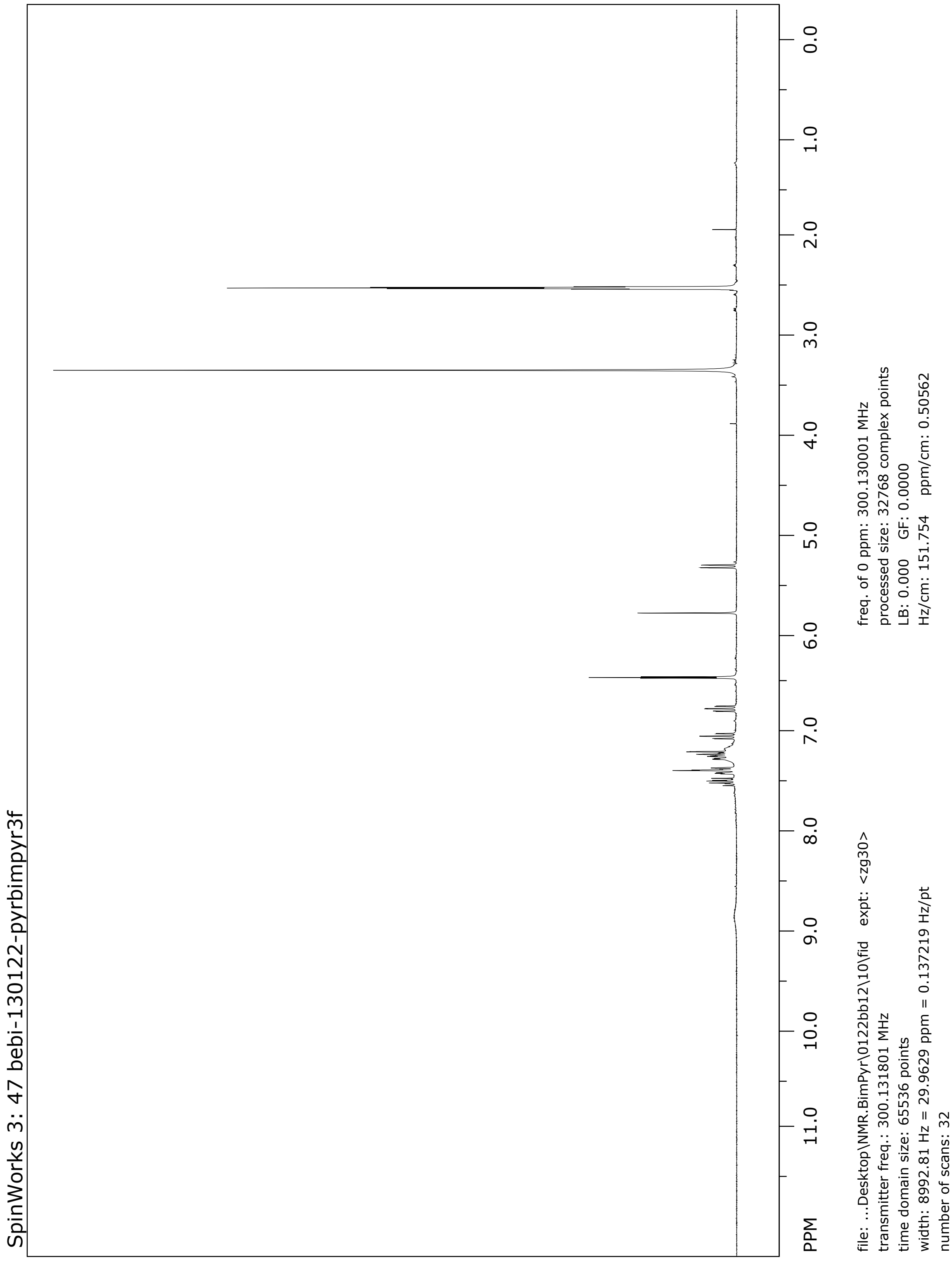

Figure S22. ^{1}H NMR spectrum of **4a**

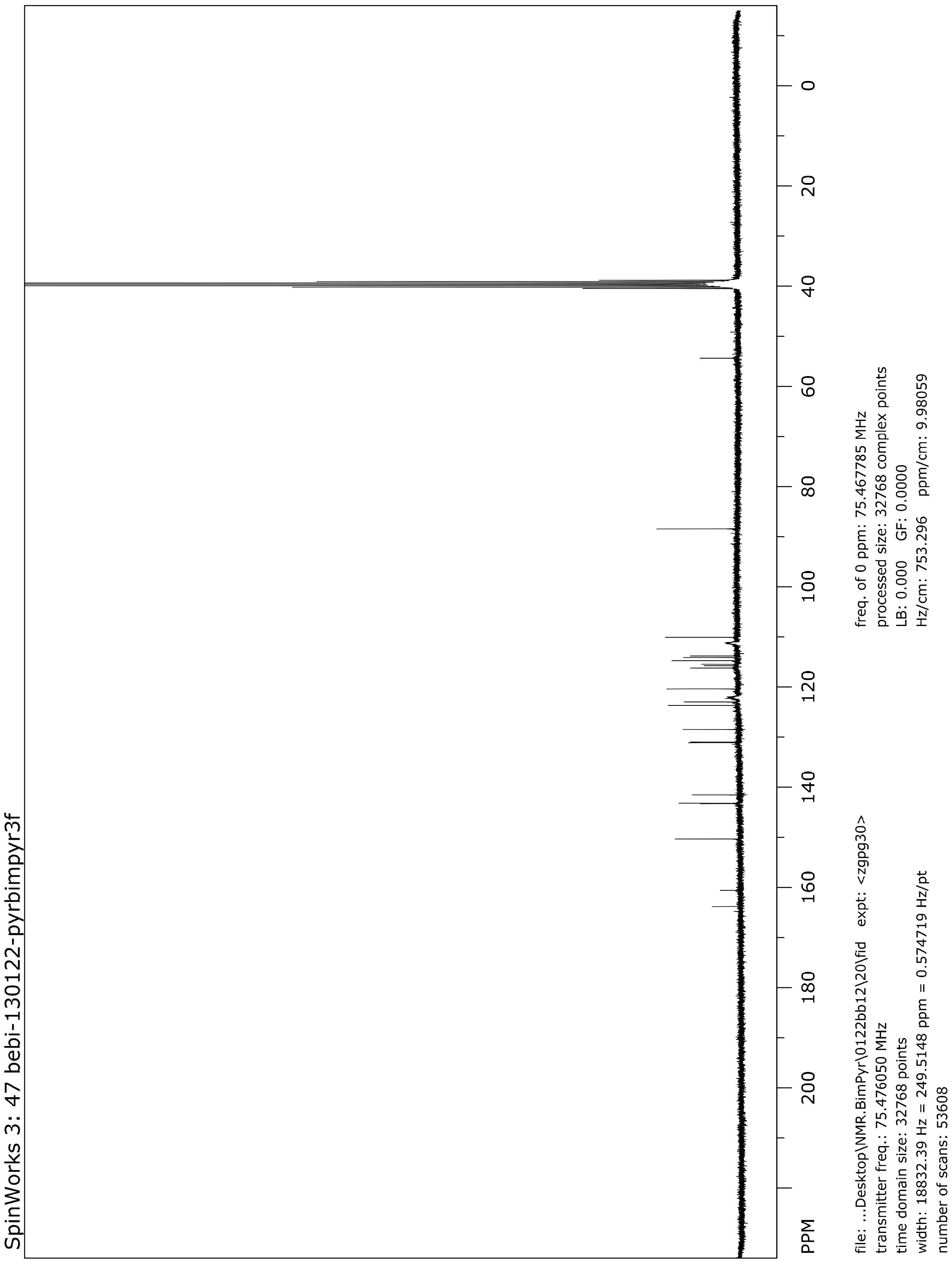

Figure S23. ^{13}C NMR spectrum of **4a**

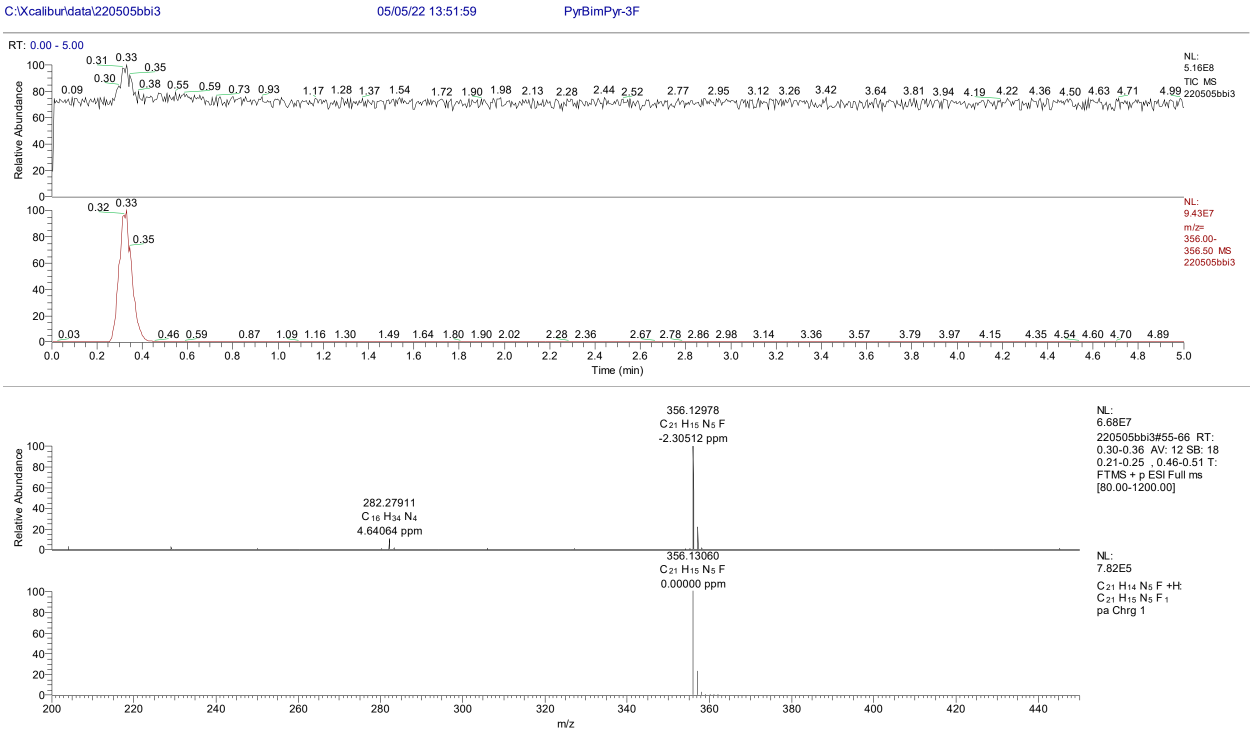

Figure S24. HRMS of **4a**

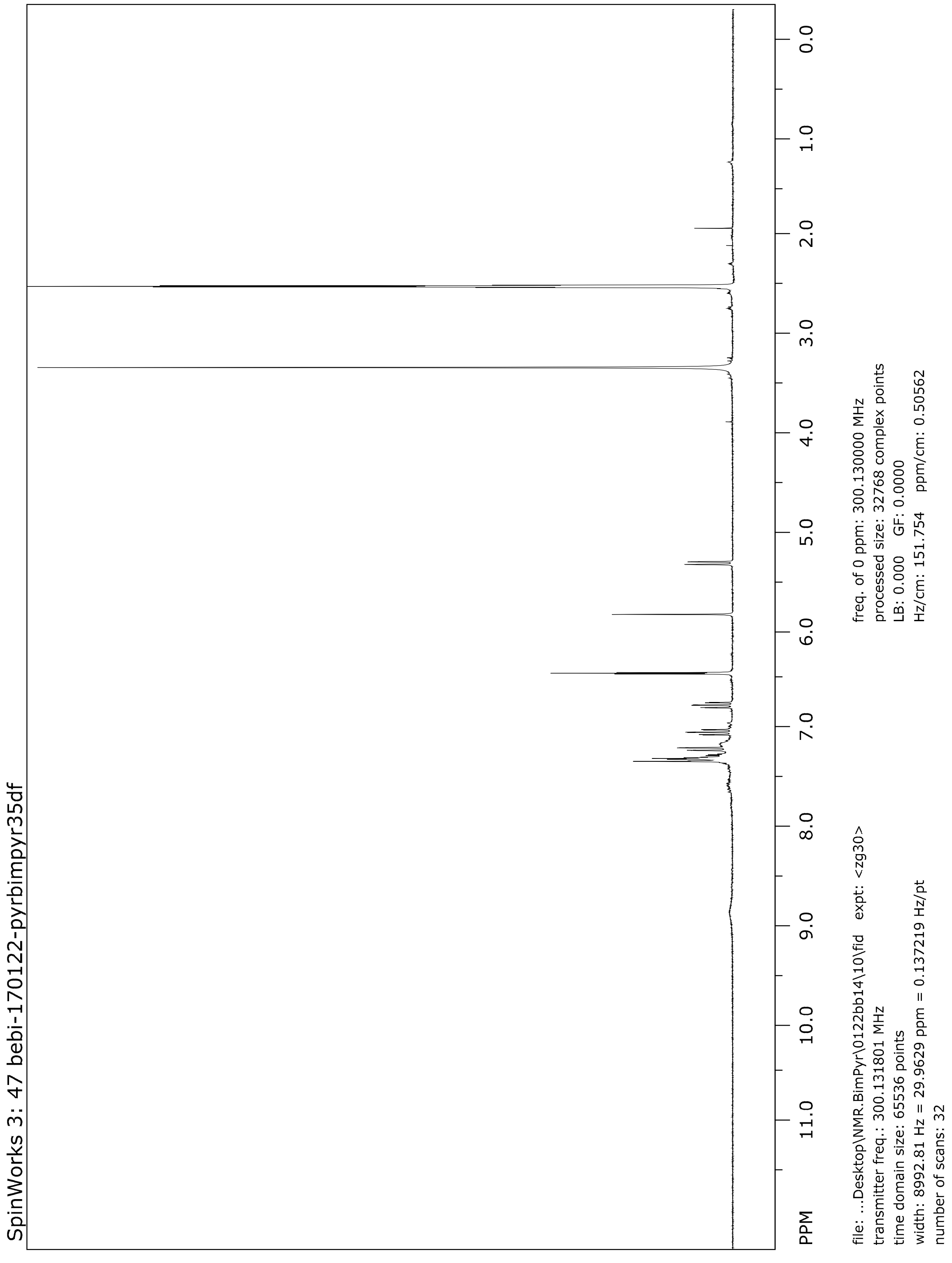

Figure S25. ^{1}H NMR spectrum of **4b**

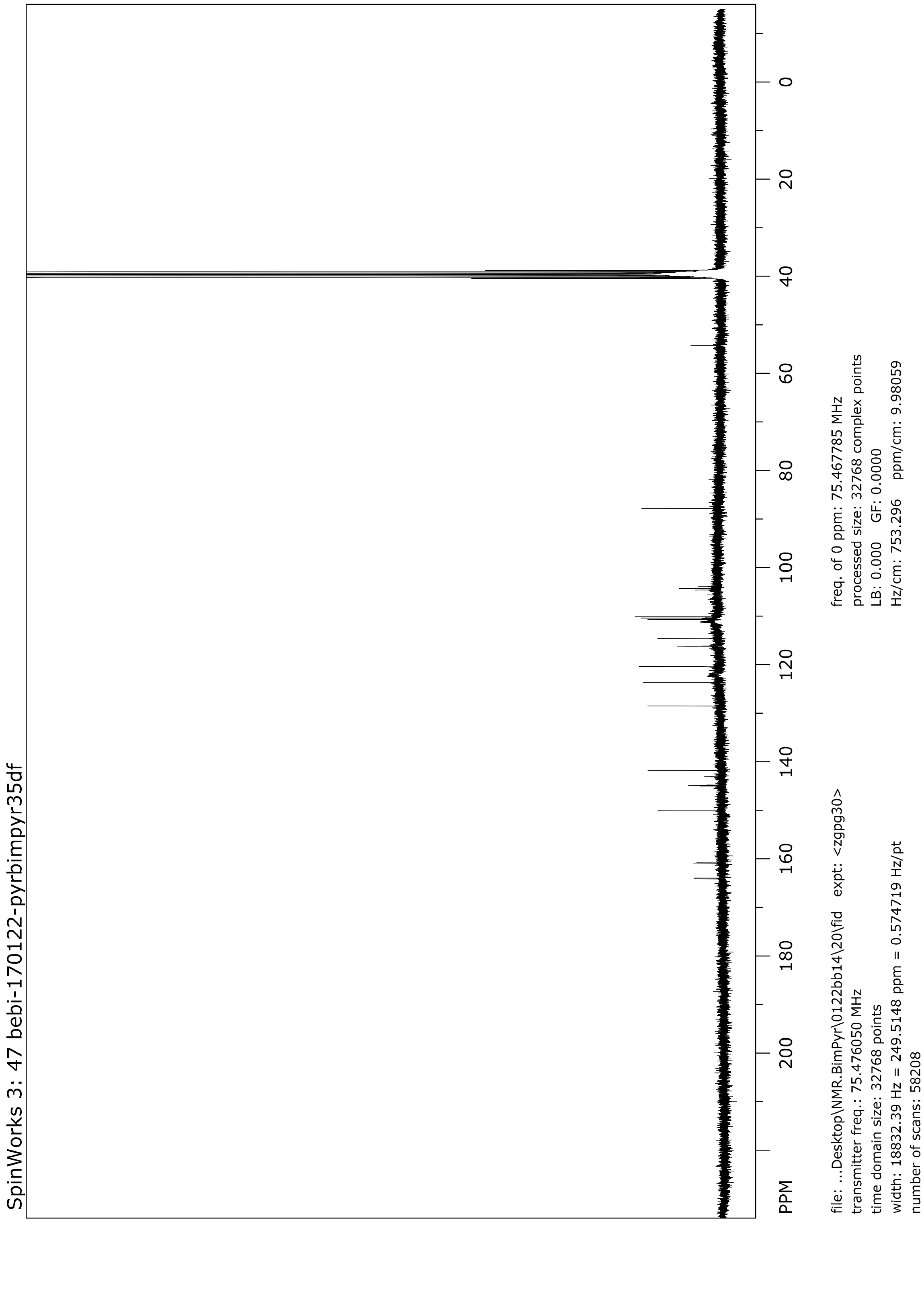

Figure S26. ^{13}C NMR spectrum of **4b**

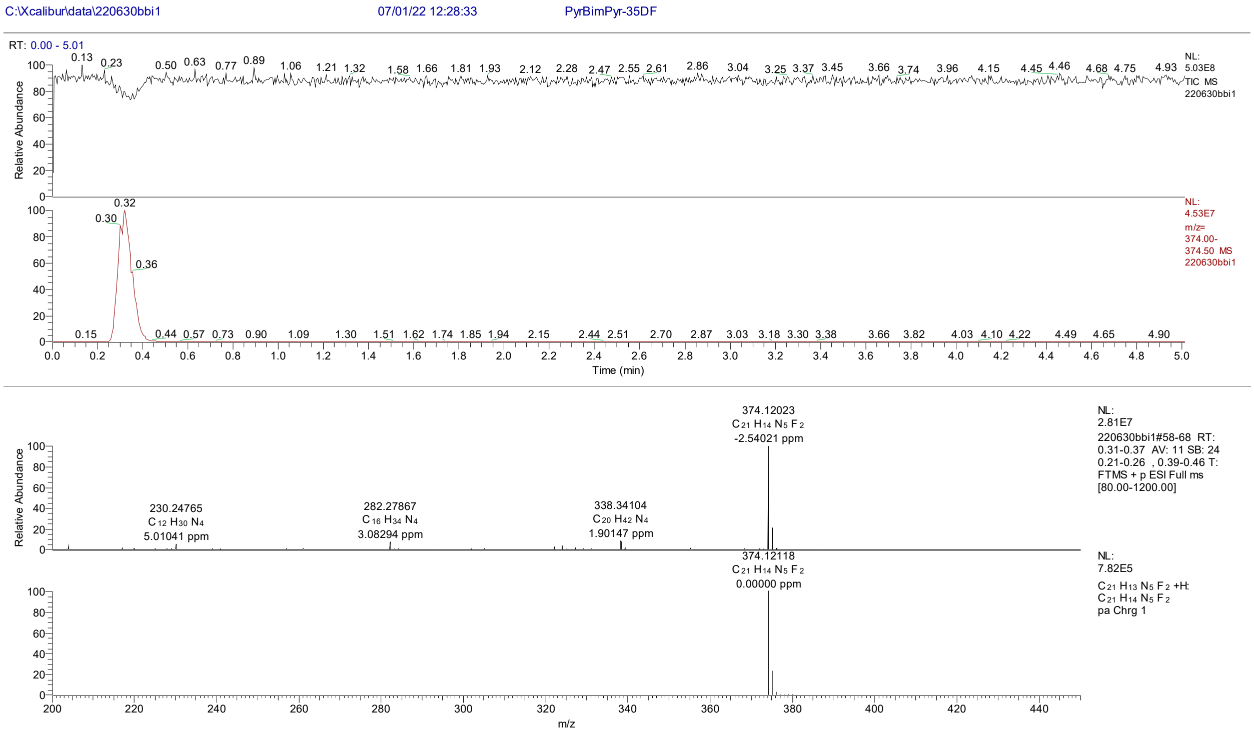

Figure S27. HRMS of **4b**

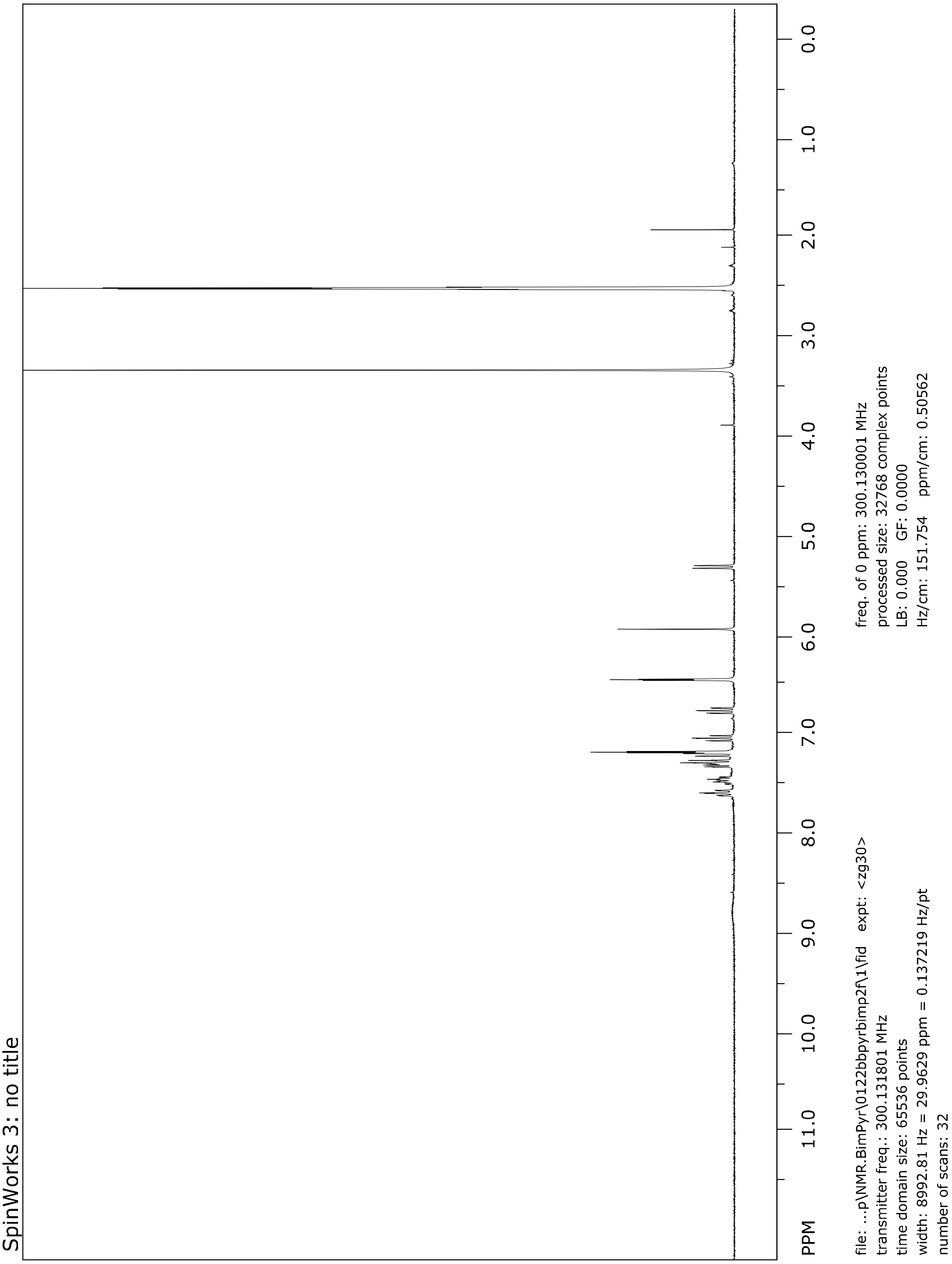

Figure S28. ^{1}H NMR spectrum of **4c**

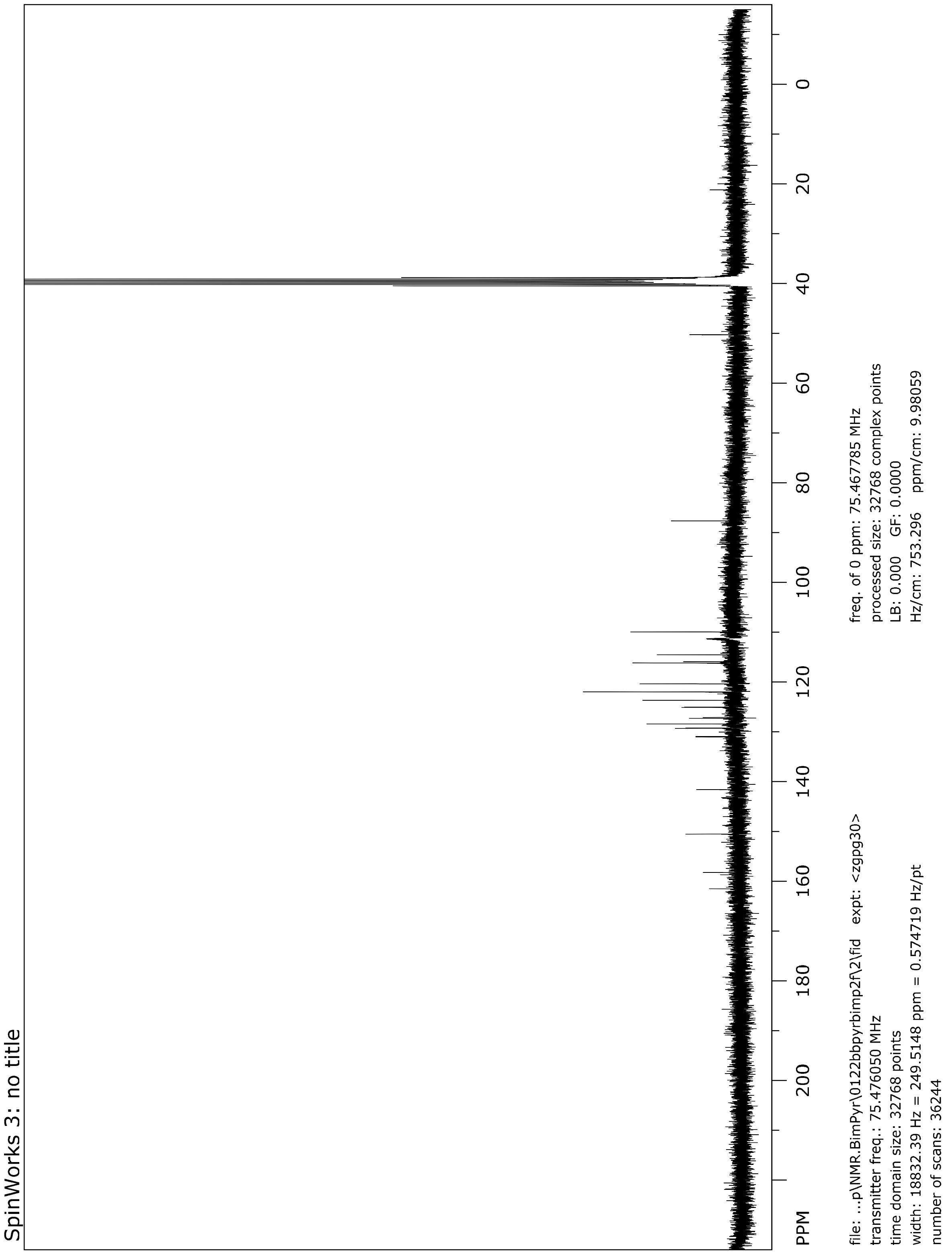

Figure S29. ^{13}C NMR spectrum of **4c**

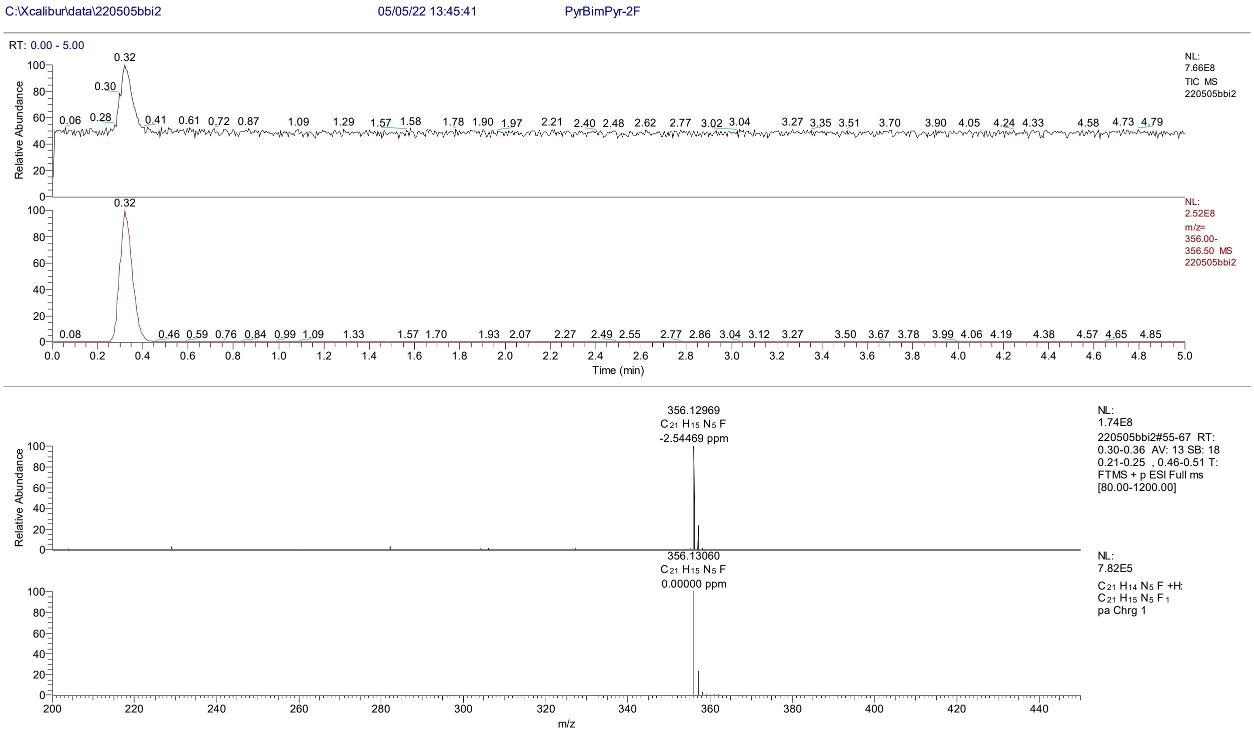

Figure S30. HRMS of **4c**

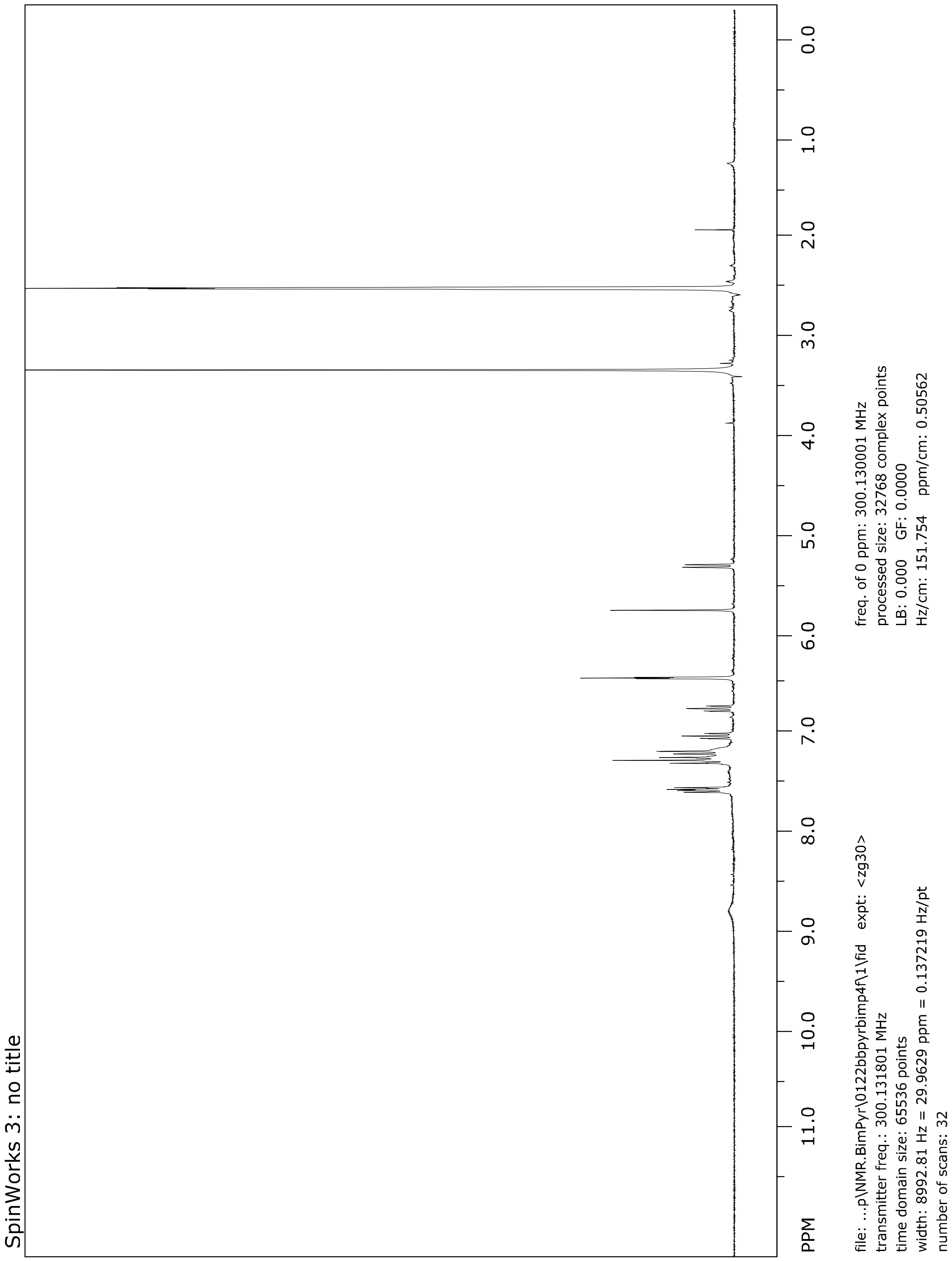

Figure S31. ^{1}H NMR spectrum of **4d**

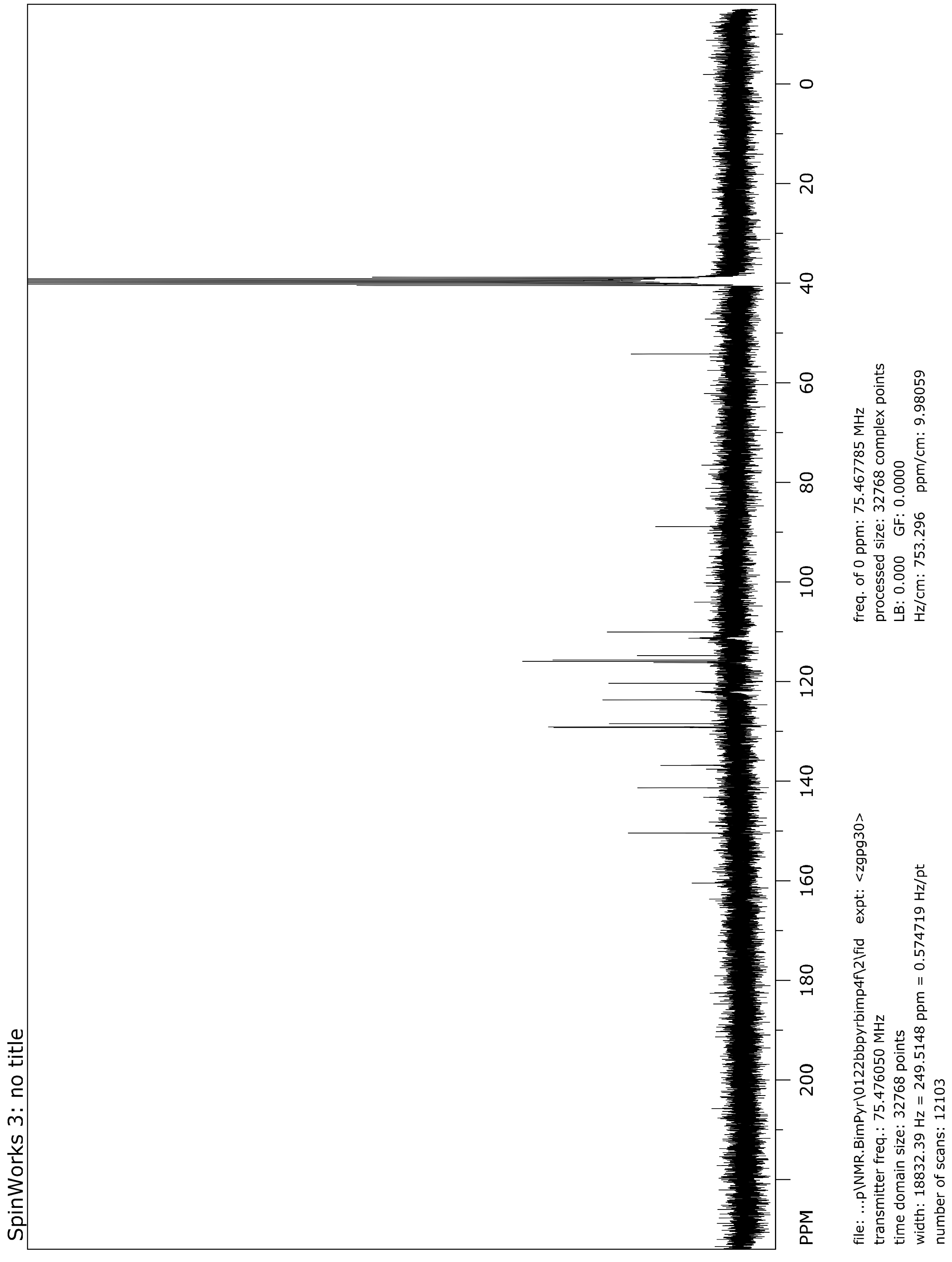

Figure S32. ^{13}C NMR spectrum of **4d**

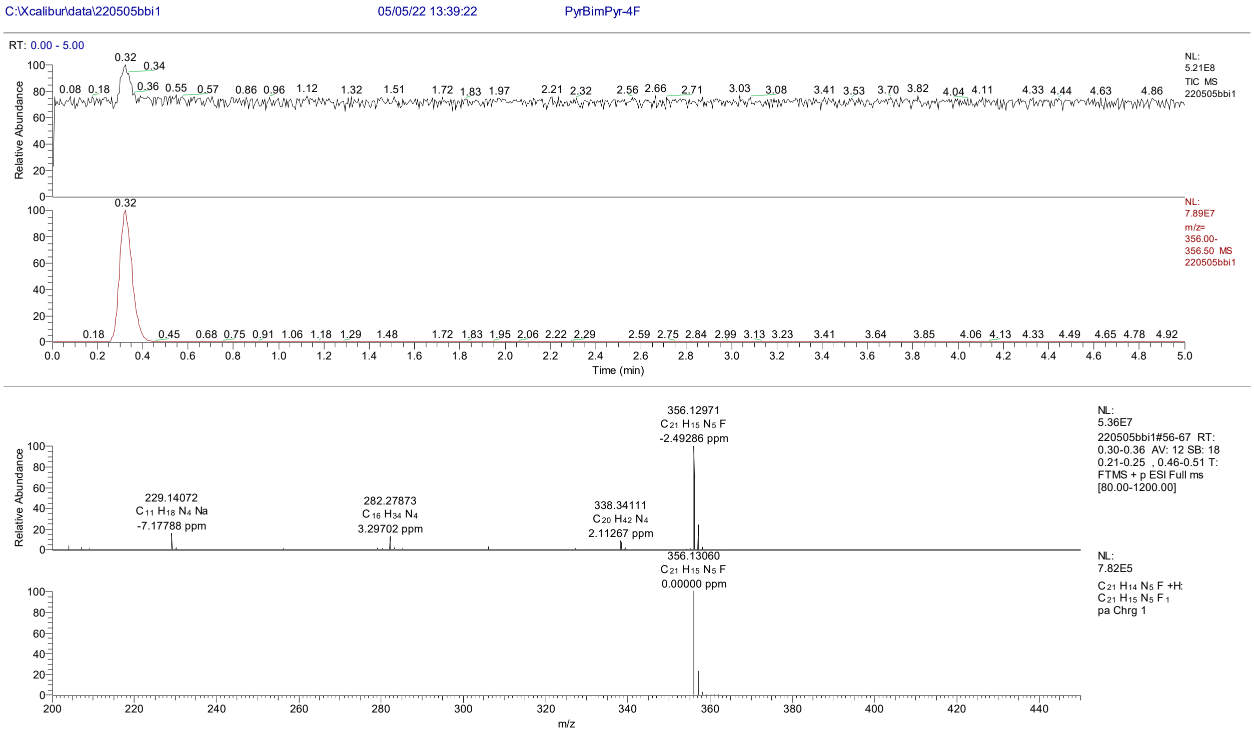

Figure S33. HRMS of **4d**
